# Chemotherapy-induced infiltration of neutrophils promotes pancreatic cancer metastasis via Gas6/AXL signalling axis

**DOI:** 10.1136/gutjnl-2021-325272

**Published:** 2022-01-12

**Authors:** Gaia Bellomo, Carolyn Rainer, Valeria Quaranta, Yuliana Astuti, Meirion Raymant, Elzbieta Boyd, Ruth Stafferton, Fiona Campbell, Paula Ghaneh, Christopher M Halloran, Dean E Hammond, Jennifer P Morton, Daniel Palmer, Dale Vimalachandran, Robert Jones, Ainhoa Mielgo, Michael C Schmid

**Affiliations:** 1 Molecular and Clinical Cancer Medicine, University of Liverpool, Liverpool, UK; 2 Molecular Physiology and Cell Signalling, University of Liverpool, Liverpool, UK; 3 Cancer Research UK Beatson Institute, Glasgow, UK; 4 Institute of Cancer Sciences, University of Glasgow, Glasgow, UK

**Keywords:** liver metastases, pancreatic cancer, macrophages, immune response

## Abstract

**Objective:**

Pancreatic ductal adenocarcinoma (PDAC) is a highly metastatic disease and cytotoxic chemotherapy is the standard of care treatment for patients with advanced disease. Here, we investigate how the microenvironment in PDAC liver metastases reacts to chemotherapy and its role in metastatic disease progression post-treatment, an area which is poorly understood.

**Design:**

The impact of chemotherapy on metastatic disease progression and immune cell infiltrates was characterised using flow and mass cytometry combined with transcriptional and histopathological analysis in experimental PDAC liver metastases mouse models. Findings were validated in patient derived liver metastases and in an autochthonous PDAC mouse model. Human and murine primary cell cocultures and ex vivo patient-derived liver explants were deployed to gain mechanistical insights on whether and how chemotherapy affects the metastatic tumour microenvironment.

**Results:**

We show that in vivo, chemotherapy induces an initial infiltration of proinflammatory macrophages into the liver and activates cytotoxic T cells, leading only to a temporary restraining of metastatic disease progression. However, after stopping treatment, neutrophils are recruited to the metastatic liver via CXCL1 and 2 secretion by metastatic tumour cells. These neutrophils express growth arrest specific 6 (Gas6) which leads to AXL receptor activation on tumour cells enabling their regrowth. Disruption of neutrophil infiltration or inhibition of the Gas6/AXL signalling axis in combination with chemotherapy inhibits metastatic growth. Chemotherapy increases Gas6 expression in circulating neutrophils from patients with metastatic pancreatic cancer and recombinant Gas6 is sufficient to promote tumour cell proliferation ex vivo, in patient-derived metastatic liver explants.

**Conclusion:**

Combining chemotherapy with Gas6/AXL or neutrophil targeted therapy could provide a therapeutic benefit for patients with metastatic pancreatic cancer.

**Significance of this study**

**What is already known on this subject?**

- Pancreatic cancer is a devastating metastatic disease for which better therapies are urgently needed.
- Pancreatic cancer frequently metastasises to the liver where the metastatic microenvironment facilitates the seeding and growth of metastases.
- Cytotoxic chemotherapy is the standard care of treatment for all patients with pancreatic cancer, including those with locally advanced or metastatic disease, and as adjuvant treatment for patients after surgical resection of their primary tumour.
- It is unclear how the metastatic microenvironment reacts to chemotherapy and its role in metastatic disease progression post-treatment.

**What are the new findings?**

- Cessation of chemotherapy induces the recruitment of neutrophils to the liver, resulting in increased metastatic growth.
- Neutrophils are recruited to the liver via CXCL1 and 2 expression by disseminated pancreatic cancer cells.
- Neutrophils recruited to the liver postchemotherapy express growth arrest specific 6 (Gas6) which leads to AXL receptor activation on tumour cells.
- Gas6-mediated activation of the AXL receptor on tumour cells promotes the regrowth of tumour cells after chemotherapy treatment in vitro and in vivo.
- Disruption of neutrophil infiltration or inhibition of the Gas6/AXL signalling axis in combination with chemotherapy inhibits metastatic growth.

**Significance of this study**

**How might it impact on clinical practice in the foreseeable future?**

- Combining chemotherapy with Gas6/AXL or neutrophil targeted therapy may offer a new opportunity in the treatment of patients with metastatic pancreatic cancer and in the adjuvant setting of patients that have undergone tumour resection.

## Background

Metastasis is the leading cause of cancer-related death. Pancreatic ductal adenocarcinoma (PDAC) frequently metastasizes to the liver^1 2^ and liver metastasis is accompanied by the formation of an inflammatory-fibrotic metastatic microenvironment that supports the colonisation and outgrowth of disseminated cancer cells.^3–6^ Myeloid immune cells, including monocytes, macrophages and neutrophils, are found in high numbers in the metastatic niche and have been shown to promote the metastatic process.^7–9^ Macrophages are highly plastic cells and, depending on their activation state, can acquire tumour supportive or tumour repressive functions.^10 11^ During liver metastasis, macrophages are prometastatic, display an immunosuppressive phenotype,^12 13^ and promote fibrosis.^3^ Emerging evidence suggests that neutrophils play a critical role during the early steps of metastasis.^14^ Neutrophils can promote the colonisation of the distant site through the release of neutrophil extracellular traps (NETs),^15 16^ induction of angiogenesis,^9 17^ secretion of leukotrienes^18^ and by their immunosuppressive activities.^19 20^ However, whether myeloid immune cell functions in pancreatic cancer metastases are altered in response to therapeutic interventions remains unknown.

Systemic spread is an early event in pancreatic cancer progression^1^ and by the time PDAC patients are diagnosed, the majority (~80%) present with non-resectable metastatic cancer.^2^ A total of 15%–20% of PDAC patients are eligible for surgical resection of their primary tumour. However, clinically undetectable micrometastatic lesions are often already present at the time the primary tumour is removed, and more than 70% relapse with distant metastasis within 24 months of surgery.^21^ The time of recurrence after surgical resection strongly correlates with overall survival, and an early hepatic metastatic relapse is associated with the worse prognosis.^22^ Following diagnosis of liver metastases, median survival on systemic chemotherapy is just 9 months.^23^

Cytotoxic chemotherapy is the standard care of treatment for all patients with pancreatic cancer, including those with locally advanced or metastatic disease and as adjuvant treatment for patients after surgical resection of their primary tumour.^24^ Gemcitabine, gemcitabine/capecitabine, nab-paclitaxel and FOLFIRINOX are the most common chemotherapeutic treatment options.^25^ Although the effect of chemotherapy on the primary tumour site is well characterised,^21^ our understanding of how chemotherapy shapes the hepatic metastatic microenvironment and how this affects metastatic disease progression remains unknown. A better understanding of this process could lead to treatments that improve the efficacy of current systemic chemotherapies.

## Results

### Gemcitabine treatment restrains metastatic progression, but disease relapses when treatment is withdrawn

**Figure 1:**
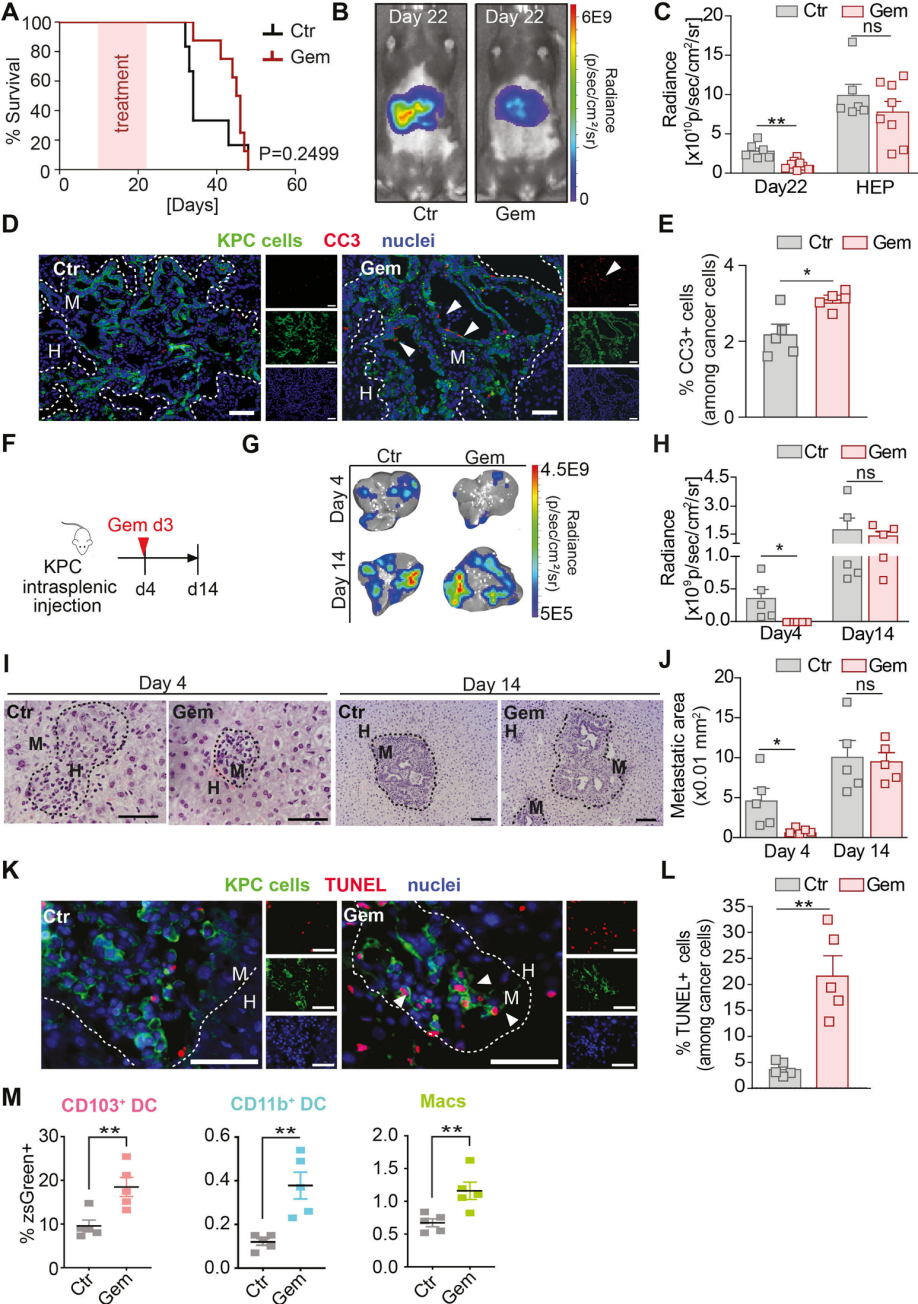
Gemcitabine restrains metastatic progression during treatment, but disease relapses and overall survival remain unchanged when treatment is withdrawn. (A–E) Liver metastasis was induced by intrasplenic implantation of 1×10^6^ KPC^luc^/^zsGreen^ cells. Starting day 12, animals were treated with gemcitabine (100 mg/kg) or control (vehicle) every 3 days with four doses in total. (A) Survival analysis of gemcitabine and control-treated mice-bearing liver metastasis; log-rank (Mantel-Cox) test, p=0.2499. Median survival for control was 22 days (n=6 mice) and gemcitabine 33.5 days (n=8 mice) after treatment initiation. (B) Representative images of bioluminescence imaging (BLI) taken 1 day after last treatment dose (day 22). (C) Tumour burden assessed by BLI in gemcitabine treated group (n=8 mice) compared with control group (n=6 mice) at day 22 and humane endpoint (HEP). (D, E) Representative immunofluorescent images (D) and quantification (E) of apoptotic KPC^luc^/^zsGreen^ cells staining positive for cleaved caspase 3 (CC3) at day 22 (n=5 mice /group). White arrowheads indicate apoptotic (CC3+) cancer cells. (F–L) Liver metastasis was induced by intrasplenic implantation of 5×10^5^ KPC^luc^/^zsGreen^ cells and animals received one dose of gemcitabine (100 mg/kg) or control (vehicle) at day 3 (F). (G, H) Representative BLI images of dissected livers (G) and change in tumour burden (H) (day 4: n=5 mice/group/time point). (I, J)Representative images of H&E-stained liver sections (I) and quantification (J). (K, L)Rrepresentative immunofluorescent images of apoptotic KPC^luc^/^zsGreen^ cells staining positive for TUNEL at day 4 (n=5 mice/group) (K) and quantification (L).White arrowheads indicate apoptotic (TUNEL+) cancer cells. (M) uptake of apoptotic zsGreen-labelled KPC FC1199^luc/zsGreen^ cancer cells by dendritic cells (DC) and macrophages (MACS) was evaluated 1 day after gemcitabine treatment. Frequency of zsGreen +cells among CD103^+^ DC, CD11b^+^ DC and MACS (n=5 mice/group). Scale bar 50 µM. Data are presented as mean±SEM. Unpaired t-test was used to calculate p values. *P<0.05; **p<0.01. H, healthy liver; M, metastases; n.s., not significant.

To model chemotherapeutic treatments of metastatic pancreatic cancer in vivo, we induced PDAC liver metastasis in mice by intrasplenic implantation of KPC derived cells and initiated gemcitabine treatment once metastatic lesions had been established (at day 12 postimplantation),^3^ (figure 1A). While KPC cancer cells were sensitive to gemcitabine when treated in vitro (online supplemental figure S1A), gemcitabine treatment did not improve the overall survival of animals with pancreatic cancer liver metastasis (figure 1A). Bioluminescent in vivo imaging analysis revealed that metastatic tumour burden was significantly reduced in the gemcitabine treated animals at the end of the treatment schedule (d22) (figure 1B), but no differences in tumour burden were detected at the humane endpoints (between day 32 and day 48 (figure 1C). H&E staining of liver tissue sections further confirmed a significant reduction of metastatic tumour lesions by the end of the treatment schedule (day 22), while this reduction was no longer detected at humane endpoints (online supplemental figure S1B–E). Postmortem analysis proofed extensive tumour burden in the liver, while tumour formation in the spleen remained minor (online supplement figure S1F, G). We next assessed tumour cell death in livers from control (saline treated) versus gemcitabine treated animals. We found that the percentage of apoptotic cancer cells, assessed by cleaved caspase 3 (CC3) staining, was significantly increased in gemcitabine treated animals compared with control tumour-bearing mice by the end of the treatment schedule (day 22) (figure 1D, E). However, by humane endpoints, after withdrawal of gemcitabine treatment, the initially observed increase in cancer cell death was lost (online supplemental figure S1H, I). Adjuvant chemotherapy is the established standard-of-care for patients who undergo surgical resection of their primary pancreatic tumour.^24^ The short time and the high frequency at which these patients relapse with metastatic disease (median of 9 months after resection^23^) strongly suggests that occult micrometastases were already established at the time of surgery.^26^ To test the effect of chemotherapy on micrometastatic lesions equivalent to the adjuvant treatment setting, we next administered a single dose of gemcitabine at day 3 post-tumour implantation, after the initial seeding period and where micro-metastatic lesions are present (figure 1F).^3 13^ Similar to what we observed with larger metastatic lesions, administration of gemcitabine also reduced micro-metastatic tumour burden (figure 1G, H) and tumour lesion areas in the liver at day 4 (figure 1I, J), but this effect was lost at day 14 (figure 1G–J). By 24 hours after gemcitabine administration, the percentage of apoptotic cancer cells (TUNEL+) in micrometastatic lesions was markedly increased in gemcitabine treated animals compared with control animals (figure 1K, L). However, at day 14, the rate of TUNEL +cancer cells declined in the gemcitabine treated tumours to similar levels as in the untreated cohort (online supplemental figure S1J, K). Macrophages and dendritic cells (DCs) are phagocytic cells from the innate arm of the immune system that play a key role in the removal of dead cells and are critical for the induction of an antitumour immune response in cancer.^27^ In order to identify the phagocytosis of cellular cancer debris by macrophages and DCs, we used flow cytometry to measure the fluorescent signal of zsGreen labelled cancer cells within macrophages (CD11b^+^F4/80^+^CD11c^neg^), CD11b^+^ DCs (CD11c^+^CD11b^+^CD103^neg^F4/80^neg^) and CD103^+^ DCs (CD11c^+^CD103^+^CD11b^neg^F4/80^neg^). All three cell populations showed a significant uptake of zsGreen signal after gemcitabine administration compared with the control group (figure 1M; online supplemental figure S1L), suggesting an activation of innate immune cells in metastatic lesions in response to chemotherapy. Taken together, these findings show that gemcitabine induces cancer cell death in PDAC metastatic lesions, but tumour growth relapses after treatment withdrawal and overall survival remains unchanged.

10.1136/gutjnl-2021-325272.supp1 Supplementary data

### Gemcitabine treatment induces a short-term activation of a proinflammatory immune response in metastatic hepatic lesions

**Figure 2:**
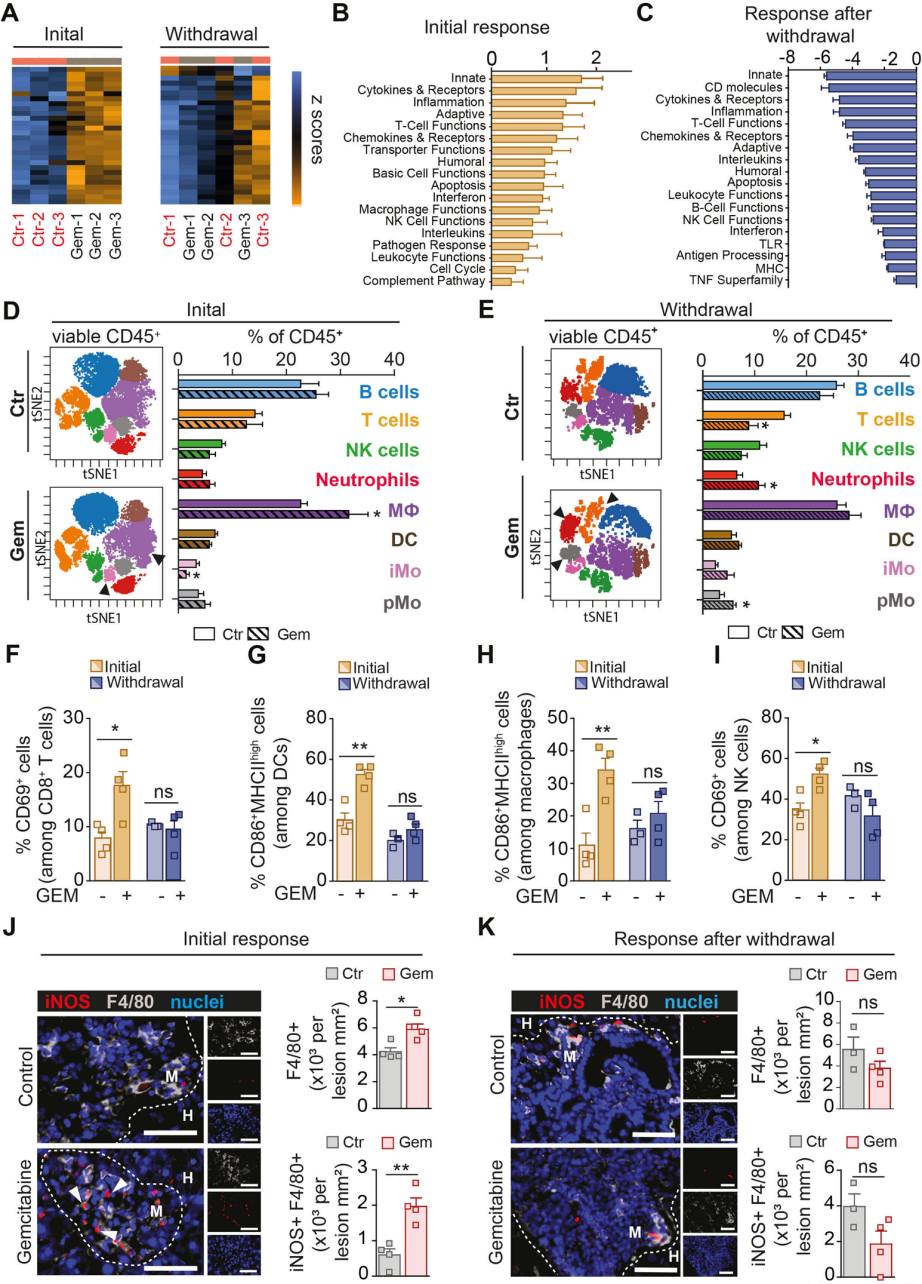
Gemcitabine administration induces a short-term activation of a proinflammatory immune response in metastatic pancreatic cancer. (A–K) Liver metastasis was induced by intrasplenic implantation of KPC^luc^/^zsGreen^ cells and animals were treated with gemcitabine (100 mg/kg) or control (vehicle) at day 3. Metastatic livers were resected at initial response (day 4) and after withdrawal of treatment (day 14) for transcriptional, mass cytometry and tissue staining analysis. (A) Heatmap depicting hierarchical clustering of pathway scores (n=3 mice/group/time point). (B–C) Graph depicting top pathway scores observed in (B) metastatic livers of gemcitabine treated animals compared with control animals during initial response (day 4) and in (C) metastatic livers after gemcitabine withdrawal (day 14) compared with the initial response (day 4). (D, E) Coloured viSNE maps with each colour representing one immune cell population assessed by mass cytometry and quantification of main immune cell types among control (CTR) and gemcitabine (GEM) treated liver metastases at day 4 (A) and day 14 (B), respectively (CTR D4 n=4 mice, GEM D4 n=4 mice; CTR d14 n=3 mice; GEM d14 n=4 mice). (F–I) Quantification of metastasis infiltrating immune cells and their activation state by mass cytometry at initial treatment response (day 4) and after treatment withdrawal (day 14). (F)Cytotoxic CD8+ T cell activation (CD69+), (G) dendritic cell (DC) activation (CD86 +MHCII^high^), (H) macrophage activation (CD86 +MHCII^high^) and (I) natural killer (NK) cell activation (CD69+) (CTR D4 n=4 mice, GEM D4 n=4 mice; CTR d14 n=3 mice; GEM d14 n=4 mice). (J, K) Representative immunofluorescent images and quantification of iNOS+ and F4/80+ macrophages in liver tumours during initial response (n=4 mice/group) (D) and after gemcitabine withdrawal (E) (n=3 mice in CTR group; n=4 mice in GEM group). White arrowheads indicate iNOS+ macrophages. Scale bar 50 µM; M=metastases, H=healthy liver. Data are presented as mean±SEM. *P<0.05; **p<0.01; n.s., not significant, by unpaired t-test. For multiple comparisons (D, E), one-way ANOVA coupled with Dunnett’s post hoc testing was performed. ANOVA; analysis of variance.

Since chemotherapy can promote the activation of an immune response in cancer^27^ we next investigated, in more detail, the immune cell activation on gene expression level in metastatic lesions during the initial response to gemcitabine treatment (day 4) and after withdrawal (day 14) using the Mouse PanCancer Immune Profiling Panel (NanoString Technologies). Hierarchical clustering of the generated pathway scores revealed that gemcitabine induces distinct transcriptional changes during the initial response, as highlighted by the separate clustering of the gemcitabine groups compared with control groups (figure 2A, left). However, the distinct signatures between control and gemcitabine-treated metastatic lesions were lost after withdrawal, as indicated by the loss of segregation between the two groups (figure 2A, right). Among the top upregulated pathways, we identified innate immune activation and T cell functions which are characteristic of an antitumour immune response (figure 2B). However, after gemcitabine withdrawal, these immune stimulatory pathways were markedly downregulated, suggesting that gemcitabine only triggers a temporal activation of an anti-tumour immune response in tumour-bearing mice (figure 2C). We next analysed disaggregated metastatic lesions by mass and flow cytometry to assess immune cell infiltration and their activation state. We found that during the initial response, macrophage numbers (CD45^+^CD11b^+^Ly6G^neg^F4/80^+^) significantly increased, and inflammatory monocytes numbers (CD45^+^CD11b^+^Ly6C^high^Ly6G^neg^F4/80^neg^) were reduced (figure 2D; online supplemental figure S2A, B). In addition, CD4^+^ T cell numbers significantly increased in response to treatment (online supplemental figure S3A, C). However, after chemotherapy withdrawal, neutrophils (CD45^+^CD11b^+^F4/80^neg^Ly6G^+^) and patrolling monocytes (pMo; CD45^+^CD11b^+^Ly6C^low^F4/80^low/neg^MHCII^neg^) increased the most in gemcitabine-treated tumours compared with control treated tumours, while T cell numbers were significantly decreased (figure 2E). The decrease in total T cell numbers was most likely due to a reduction in CD8^+^ T cells, since the less abundant CD4^+^FoxP3^+^ T regulatory cells (T_regs_) rather increased (online supplemental figure S3A–D). Consistent with an antitumour immune response, we found a significant increase in the activation of CD8^+^ and CD4^+^ T cells (figure 2F; online supplemental figure S3E, F), DCs (figure 2G), macrophages (figure 2H) and NK cells (figure 2I) during the initial response to gemcitabine (online supplemental figure S2A, B). Again, this effect was lost after withdrawal of the treatment.

Further analysis of metastatic liver tissues confirmed that gemcitabine treatment induces the overall accumulation of macrophages (F4/80^+^), particularly of macrophages with a proinflammatory phenotype (iNOS^+^) (figure 2J) while macrophages with an immunosuppressive phenotype (CD206^+^ and YM1^+^) were reduced (online supplemental figure S3G-J). However, no significant changes in macrophage infiltration or activation were observed after treatment withdrawal (figure 2K; online supplemental figure S3K, L). Taken together these data suggest that gemcitabine administration induces the activation of an antitumourigenic immune response at the metastatic site, characterised by an increase in proinflammatory macrophages, activated CD8^+^ T cells and NK cells. However, after treatment withdrawal the initial immune cell activation is lost and metastatic lesions revert back to an immunosuppressive microenvironment, which is commonly found in established metastatic PDAC tumours.^12 13^

### Macrophage depletion after gemcitabine treatment increases CD8+ T cell infiltration, but neutrophil depletion has no effect on CD8+ T cell numbers

**Figure 3:**
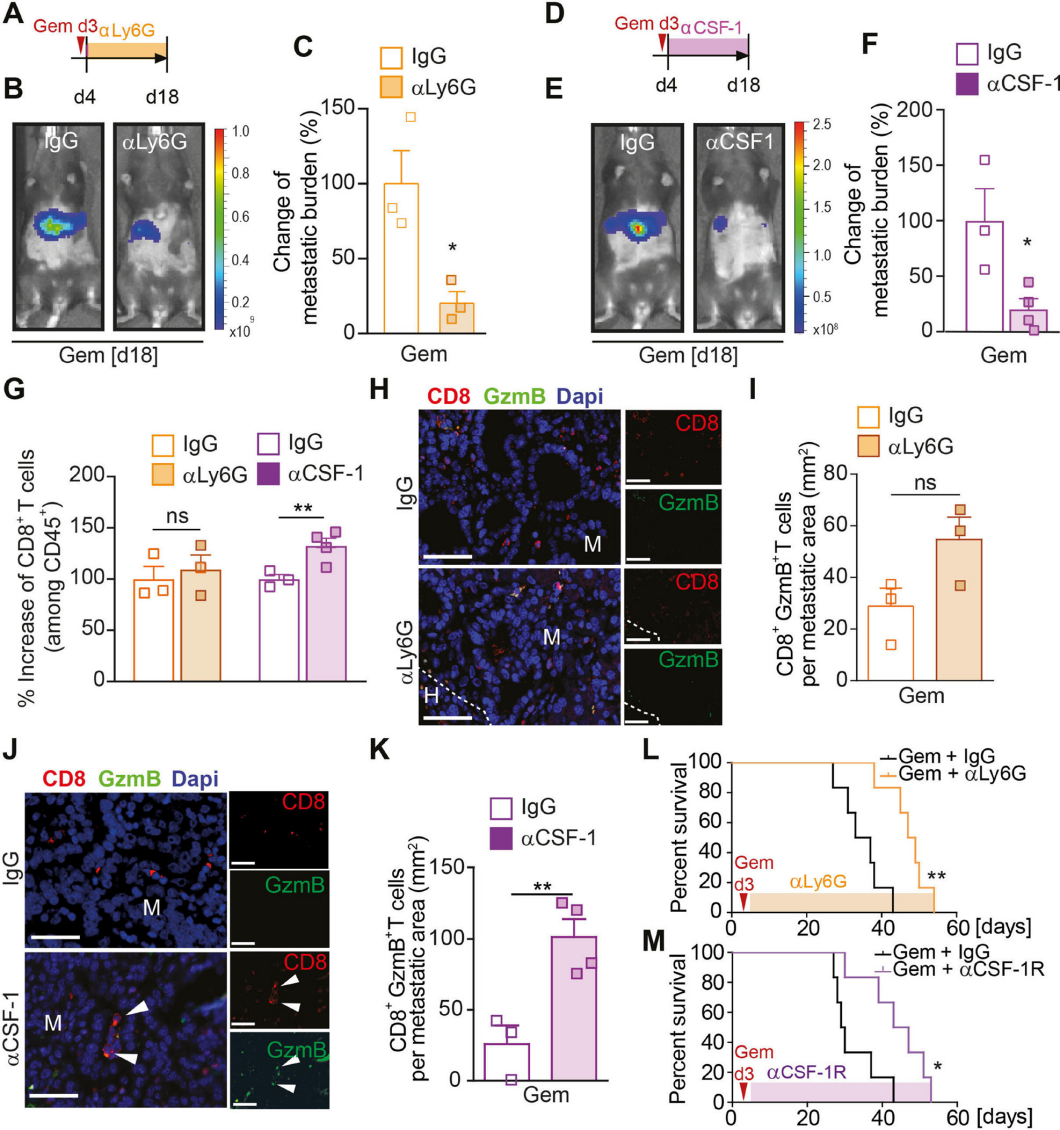
Macrophage depletion increases CD8+ T cell infiltration, but neutrophil depletion has no effect on CD8+ T cell numbers. (A–M) Liver metastasis was induced by intrasplenic implantation of KPC^luc^/^zsGreen^ cells and animals were treated with gemcitabine (100 mg/kg) or control (vehicle) at day 3. (A–C, G–I) At day 4, mice were treated with IgG control (CTR) or αLy6G antibody. Schematic illustrating experiment (A). Change in tumour burden was quantified by in vivo BLI (n=3 mice/group). Representative images (B) and quantification (C). (D–G, J, K) At day 4, mice were treated with IgG control (CTR) or αCSF-1 antibody. Schematic illustrating experiment (D). Change in tumour burden was quantified by in vivo BLI (CTR n=3 mice; αCSF-1 n=4 mice). Representative images (E) and quantification (F). (G) Change in CD8+ T cell infiltration into metastatic lesions was quantified by flow cytometry analysis in mice treated with αLy6G or αCSF-1 or their corresponding IgG controls. (H, I) Representative immunofluorescent images of CD8+GranzymeB+ T cell staining of liver sections from mice treated with IgG or αLy6G (H) and quantification (I) of CD8+GranzymeB+ T cells (GranzymeB=GzmB). (J, K) Representative immunofluorescent images of CD8+GranzymeB+ T cell staining of liver sections from mice treated with IgG or αCSF-1 (J) and quantification (K) of CD8+GranzymeB+ T cells. White arrowheads indicate CD8+GranzymeB+ T cells. (L) Liver metastasis was induced by intrasplenic implantation of 1×10^6^ KPC^luc^/^zsGreen^ cells. At day 3, all animals were treated with gemcitabine (100 mg/kg). At day 4, mice were treated with IgG control (CTR) or αLy6G antibody. survival analysis of gemcitabine + IgG and gemcitabine + αLy6G antibody-treated mice-bearing liver metastasis; log-rank (Mantel-Cox) test, p=0.0022. Median survival for gemcitabine + IgG was 35 days (n=6 mice) and gemcitabine + αLy6G 48 days (n=6 mice). (M) same as (L), but at day 4, mice were treated with IgG control (CTR) or αCSF-1R antibody. Survival analysis of gemcitabine + IgG and gemcitabine + αCSF-1R antibody-treated mice-bearing liver metastasis; log-rank (Mantel-Cox) test, p=0.0168. Median survival for gemcitabine + IgG was 29.5 days (n=6 mice) and gemcitabine + αCSF-1R 45 days (n=6 mice). Scale bar 50 µM. Data are presented as mean±SEM. Unpaired t-test was used to calculate p values. *P<0.05; **p<0.01. H, healthy liver; M, metastases; n.s., not significant.

Since neutrophils and macrophages can both effectively suppress CD8^+^ T cell responses,^11 14^ we next questioned whether the depletion of either of these myeloid cell types is sufficient to stop metastatic relapse and to sustain the initially observed CD8^+^ T cell response (figure 2B and F). To address this question, we ran two separate depletion studies using monoclonal antibodies targeting neutrophils (αLy6G) or macrophages (αCSF-1) in the presence or absence of gemcitabine treatment. Liver metastasis was induced by intrasplenic implantation of KPC cells. After 3 days, mice-bearing micrometastatic lesions were treated with gemcitabine or saline (control) and 1 day later (day 4) we commenced the depletion of neutrophils (figure 3A–C) or macrophages (figure 3D–F) using αLy6G and αCSF-1 antibodies, or their corresponding isotype controls (IgG). Depletion of neutrophils after gemcitabine administration markedly reduced the metastatic tumour burden compared with gemcitabine/IgG treatment (figure 3B, C, online supplemental figure S4A, B), but depletion of neutrophils in the absence of gemcitabine did not have any effect on metastatic tumour burden (online supplemental figure S4C). In contrast, depletion of macrophages by αCSF-1 significantly reduced metastatic tumour burden in both saline (control) and gemcitabine treated mice (figure 3E, F; online supplemental figure S4D, E). Flow cytometry analysis of disaggregated metastatic lesions derived from gemcitabine treated animals revealed that the depletion of macrophages increased CD8^+^ T cell infiltration, while neutrophil depletion did not affect CD8^+^ T cell infiltration (figure 3G). In agreement with these findings, we did not detect an increase in Granzyme B expression in CD8^+^ T cells in gemcitabine-treated animals where neutrophils were depleted (figure 3H), but we found a significant increase of Granzyme B expressing CD8^+^ T cells in metastatic lesions of gemcitabine-treated mice where macrophages were depleted (figure 3J, K). We also confirmed that applied macrophage-depletion and neutrophil-depletion strategies indeed reduced their corresponding numbers at the metastatic site (online supplemental figure S4F–H). Notably, neutrophil-depletion or macrophage-depletion after gemcitabine treatment also increased overall survival of the mice compared with gemcitabine treatment alone (figure 3L, M). Taken together, these data show that gemcitabine administration is accompanied by an infiltration of macrophages during the initial response, while neutrophils are recruited to the metastatic site after therapy withdrawal. Depletion of macrophages or neutrophils after gemcitabine withdrawal enhances the therapeutic effect of gemcitabine. Notably, while macrophage depletion restores CD8^+^ T cell infiltration and activation, neutrophil depletion does not affect CD8^+^ T cells, suggesting that neutrophils promote metastatic relapse in a CD8^+^ T cell independent manner.

### Chemotherapy withdrawal triggers the recruitment of Gas6-expressing neutrophils to hepatic metastatic tumours

**Figure 4:**
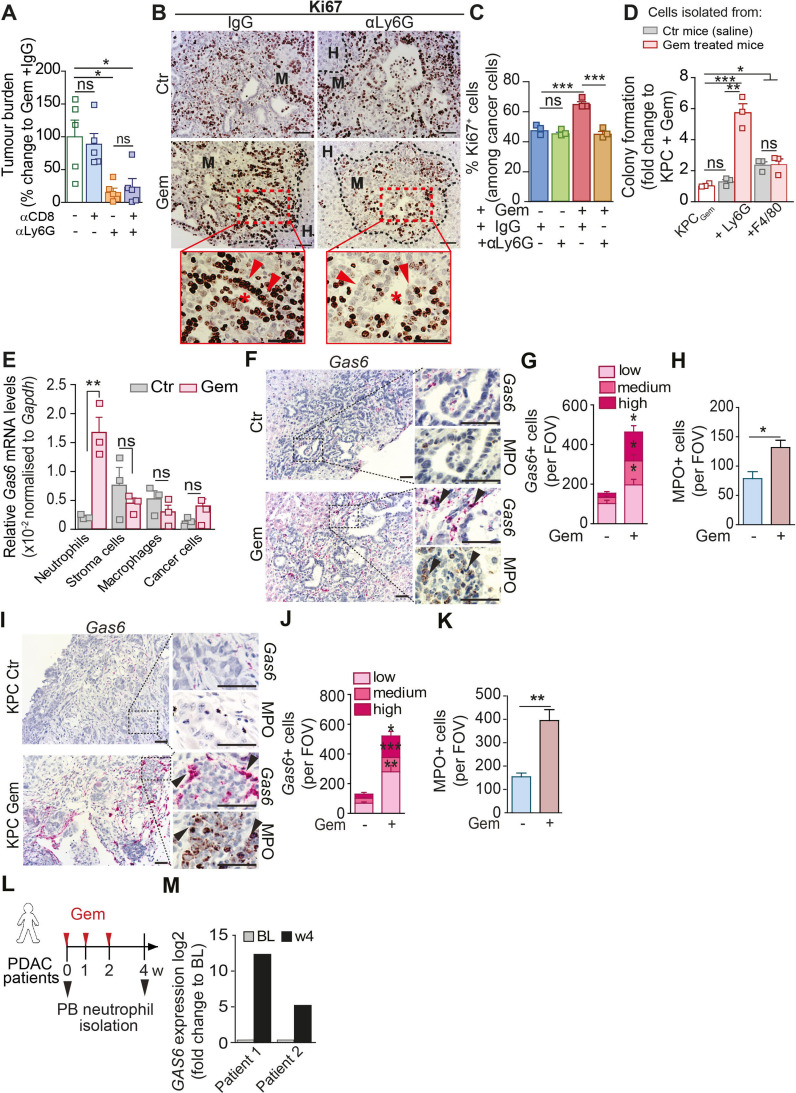
Neutrophils promote cancer cells proliferation and Gas6 is highly expressed by metastatic associated neutrophils after gemcitabine treatment. (A) Liver metastasis was induced by intrasplenic implantation of KPC^luc^/^zsGreen^ cells and animals were treated with gemcitabine (GEM; 100 mg/kg) at day 3. At day 4, mice were treated with αLy6G or IgG controls for 2 weeks; at day 7, mice were treated with αCD8 or IgG controls until end point (day 14). Change in tumour burden was quantified by ex vivo bioluminescence imaging (BLI) (n=5 mice/group). (B–C) Liver metastasis was induced by intrasplenic implantation of KPC^luc^/^zsGreen^ cells. Mice were treated with gemcitabine or saline 3 days postcell implantation. Treatment with αLy6G or control IgG started at D4 (n=4 mice/group). Livers were resected after 14 days and assessed by Ki67 staining (proliferation marker). Representative IHC images (B) and quantification of proliferating Ki67+ tumour cell frequency in metastatic livers (C). Inset: asterisks indicate ductal structures formed by metastatic tumour cells (red arrow head). (D)Colony formation assay of gemcitabine stressed KPC cells in the presence or absence of metastasis infiltrating neutrophils (+Ly6G) or macrophages (+F4/80) isolated from tumour-bearing livers of mice at day 14 after treatment with GEM or saline treated (CTR). Bar graph shows fold upregulation of BLI signal compared with Gem-treated KPC cells alone (red shaded) (three independent experiments; mean±SEM). (E) Quantification of *Gas6* mRNA levels by real time PCR in intrametastatic pancreatic cancer cells, neutrophils (Ly6G), macrophages (F4/80) and non-immune stromal cells (zsGreen^neg^CD45^neg^), isolated by fluorescence activated cell sorting from established metastatic livers at day 14 after treatment with GEM or untreated (CTR). Bar graph shows relative expression of *Gas6* in cells derived from GEM-treated mice and untreated mice (data are from three independent experiments; mean±SEM). (F–H) Representative images (F) of myeloperoxidase (MPO) and *Gas6* staining using RNAscope in serial sections from metastatic livers derived from untreated (CTR) or GEM treated mice (n=3 mice/group). Arrowheads indicate *Gas6+* staining in neutrophil-rich areas. Scoring of *Gas6* signal per field of view (G) and MPO staining quantification (H). (I–K) Metastatic tumours in livers of the spontaneous mouse pancreatic cancer model Kras^G12D^;Trp53^R172H^;Pdx1-Cre (KPC mice) treated with Gemcitabine (KPC Gem) or left untreated (KPC Ctr) were isolated and analysed (n=3 mice/group). Representative images (I) of MPO and *Gas6* staining using RNAscope in serial sections from metastatic tissue sections. Arrowheads indicate *Gas6+* staining in neutrophil-rich areas. (J) Scoring of Gas6 signal per field of view and (K) MPO staining quantification. (L, M) Peripheral blood neutrophils were isolated from metastatic PDAC patients during their first cycle of gemcitabine treatment and *GAS6* mRNA levels were assessed by real time PCR. Schematic illustration of treatment regimen and patient blood sample collection (L). Quantification of data (M) (BL=baseline, prior treatment) (n=2 patients). Scale bar=50 µM. Data are presented as mean±SEM. Unpaired t-test or ANOVA with Bonferroni was used to calculate p values. *P<0.05; **p<0.01; ***p<0.001. ANOVA; analysis of variance; H, healthy liver; IHC, immunohistochemistry; M, metastases; n.s., not significant; PDAC, pancreatic ductal adenocarcinoma.

Next, we further explored the mechanism by which neutrophils promote metastatic relapse after chemotherapy withdrawal. Neutrophil depletion after gemcitabine treatment reduced metastases in the presence and absence of CD8^+^ T cells (figure 4A; online supplemental figure S4I, J), suggesting that the neutrophils can directly affect cancer cell regrowth. Hence, we next assessed cancer cell proliferation in tumour sections after gemcitabine withdrawal (day 14). After gemcitabine withdrawal, metastatic deposits showed a significant increase of proliferating (Ki67^+^) cancer cells compared with metastatic deposits from the saline treated control group (figure 4B, C). Importantly, the depletion of neutrophils only reduced cancer cell proliferation associated with gemcitabine withdrawal, and had no impact on cancer cell proliferation in saline (control) treated mice, suggesting a treatment induced growth promoting function of neutrophils (figure 4B, C). Thus, to test this hypothesis, we isolated metastasis infiltrating neutrophils from metastatic livers from mice treated with saline (control) or gemcitabine, and cocultured them with gemcitabine pretreated pancreatic cancer cells under anchorage independent growth conditions ex vivo. Gemcitabine-treated pancreatic cancer cells were unable to form colonies (figure 4D; online supplemental figure S4K). Strikingly, coculturing of neutrophils isolated from gemcitabine treated metastatic livers with gemcitabine-treated cancer cells enabled the cancer cells to grow and form colonies, while neutrophils isolated from control (saline treated) metastatic lesions were unable to promote cancer cell proliferation (figure 4D). In contrast, while metastases derived macrophages were also able to significantly increase cancer cell colony formation, the macrophage-growth promoting functions were Gas6-independent, unaffected by gemcitabine and markedly less potent compared with neutrophils derived from gemcitabine treated mice (figure 4D; online supplemental figure S4L). In agreement with these findings, in vivo, Ki67^+^ cancer cell numbers were reduced in macrophage depleted mice independent of their treatment (online supplemental figure S4M, N). Taken together, these data show that gemcitabine treatment makes neutrophils acquire a promitogenic capacity that promotes cancer cell proliferation.

We next aimed to understand how neutrophils promote cancer cell proliferation. To achieve this goal, we performed RNA sequencing of metastasis infiltrating neutrophils isolated from saline treated metastatic livers (Ly6G_Ctr_) and gemcitabine treated metastatic livers (Ly6G_Gem_) (online supplemental figure S4O). Differently expressed genes were first filtered for GO terms extracellular and receptor ligand activity. Among the resulting n=141 genes, we identified Growth Arrest Specific protein 6 (Gas6) as one of the top upregulated genes in neutrophils derived from gemcitabine treated metastatic lesions compared with control metastatic lesions (online supplemental tables S1, S2). Gas6 and its main receptor AXL are overexpressed in pancreatic cancer and their expression correlates with poor prognosis.^28 29^ Gas6/AXL signalling in cancer cells is associated with tumour cell proliferation, epithelial mesenchymal transition and metastases.^30 31^ Subsequent analysis of *Gas6* expression in flow cytometry sorted neutrophils, non-immune stroma cells, macrophages and cancer cells confirmed that within the metastatic tumour microenvironment, neutrophils markedly upregulate *Gas6* expression in response to gemcitabine treatment and neutrophils are the main source of *Gas6* after gemcitabine withdrawal (figure 4E; online supplemental figure S5A).

10.1136/gutjnl-2021-325272.supp2 Supplementary data

10.1136/gutjnl-2021-325272.supp3 Supplementary data

In agreement with these findings, we found a marked upregulation of Gas6 expression levels in neutrophil-rich areas proximate to cancer cells after gemcitabine withdrawal in serial tissue sections derived from experimental (figure 4F–H; online supplemental figure S5B, D) and spontaneous hepatic metastatic lesions (figure 4I–K; online supplemental figure S5C, D). In contrast, chemotherapy withdrawal did not increase Gas6 levels in metastatic livers from neutrophil-depleted mice (online supplemental figure S5E, F). Moreover, we only found an increase of Gas6 expressing neutrophils in tumour-bearing livers, but not in tumour-free lung tissues, suggesting Gas6 expressing neutrophils preferentially accumulate at the metastatic tumour site (online supplemental figure S5G, H).

**Figure 5:**
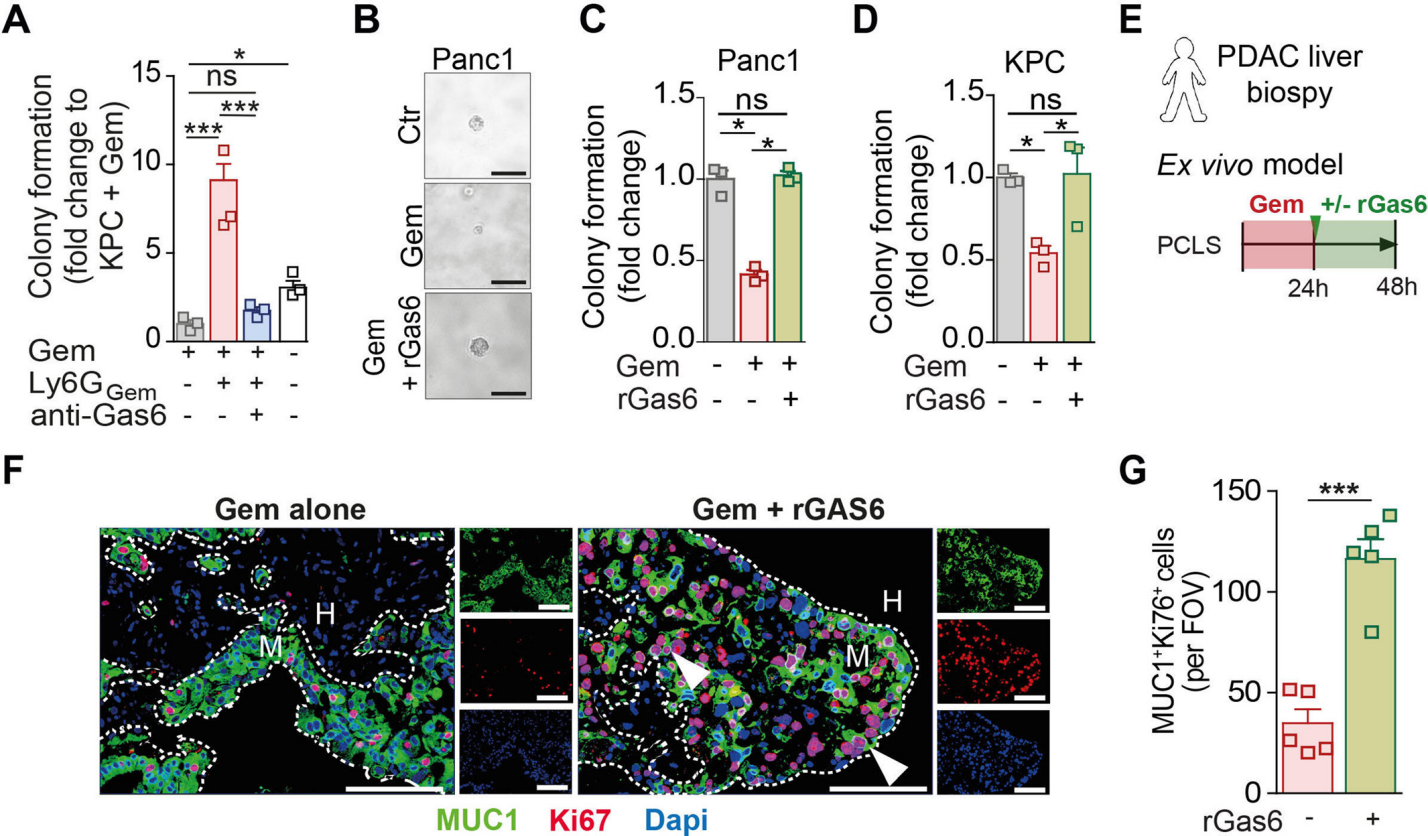
Gas6 is necessary for neutrophil-mediated cancer cell regrowth after gemcitabine treatment. (A) Quantification of colony formation assay of gemcitabine treated KPC^luc/zsGreen^ cells in the presence or absence of Gas6 neutralising antibody (anti-Gas6) with or without metastasis infiltrating neutrophils (Ly6G_Gem_) isolated from mice treated with gemcitabine. Bar graph shows fold upregulation of bioluminescence imaging (BLI) signal compared with gemcitabine-treated KPC^luc/zsGreen^ cells alone (three independent experiments; mean±SEM). (B–D) Colony formation assay of gemcitabine-treated human Panc1 and murine KPC^luc/zsGreen^ cells in the presence or absence or recombinant Gas6 (rGas6). (B) Representative images of Panc1 colonies. (C) Quantification of colony numbers (fold change compared with untreated Panc1 cells) (three independent experiment; mean±SEM). (D) Quantification of BLI signal from KPC^luc/zsGreen^ colonies (fold change compared with untreated KPC cells) (three independent experiments; mean±SEM). (E–G) Schematic illustration of experiment (E): Human precision cut liver slices (hPCLSs) were initially treated with gemcitabine for 24 hours then cultured in the presence or absence of rGas6 for the following 24 hours. hPCLSs were assessed by MUC1 (cancer cell marker) and Ki67 immunofluorescent staining (proliferation marker). (F) Representative if images and (G) quantification of proliferating Ki67+ tumour cell frequency in ex vivo treated hPCLS (n=5 patient biopsies). Arrowheads indicate Ki67+ cancer cells. Scale bar 50 µM. data are presented as mean±SEM. Unpaired t-test or ANOVA with Bonferroni was used to calculate p values. *P<0.05; **p<0.01; ***p<0.001. ANOVA, analysis of variance; H, healthy liver; M, metastases; n.s., not significant.

We observed the same changes when we treated metastatic tumour-bearing mice with nab-paclitaxel or FOLFIRINOX, both commonly used chemotherapy regimens in PDAC^25^ (online supplemental figure S5I). With all chemotherapeutic treatments, metastatic tumour burden temporarily decreased but was followed by metastatic relapse (online supplemental figure S5J) which was accompanied by an influx of Gas6-expressing neutrophils into metastatic lesions (online supplemental figure S5K–N). These results suggest that the increased accumulation of Gas6-expressing neutrophils in relapsed metastatic lesions after chemotherapy treatment occurs in response to different chemotherapeutic treatment regimens and is therefore not agent specific. We next assessed Gas6 expression in circulating neutrophils in patients with metastatic pancreatic cancer and in our mouse metastases model. We collected patient blood samples prior (baseline) and after (week 4) their first cycle of gemcitabine treatment (figure 4L). We found that Gas6 expression increased in circulating neutrophils 4 weeks after the first dose of treatment (figure 4M; online supplemental figure S6A). Similarly, in the preclinical mouse model, Gas6 expression was increased in circulating murine neutrophils after gemcitabine withdrawal (online supplemental figure S6B). Since the release of NETs by apoptotic neutrophils has been shown to promote pulmonary metastatic outgrowth in breast cancer models,^16^ we also analysed the presence of apoptotic (TUNEL+) neutrophils in liver metastases. However, we could only detect a few apoptotic neutrophils within liver metastases and their numbers remained unaffected by gemcitabine treatment (online supplemental figure S6C, D). To assess the biological importance of Gas6 in promoting regrowth of metastatic cancer cells, we next isolated metastasis infiltrating neutrophils from gemcitabine-treated tumour-bearing mice (Ly6G_Gem_) and cocultured those neutrophils with gemcitabine treated cancer cells in the presence or absence of a Gas6 neutralising antibody. We found that Gas6 secretion from neutrophils promotes cancer cell regrowth, in fact, the addition of a neutralising Gas6 antibody abolished the promitogenic effect of neutrophils (figure 5A). Next, we tested whether Gas6 is sufficient to promote the regrowth of gemcitabine treated cancer cells. We pretreated human Panc1 cells and mouse derived KPC cells with gemcitabine and measured regrowth of the cancer cells in the presence or absence of recombinant Gas6. Addition of recombinant Gas6 was sufficient to promote the regrowth of gemcitabine treated human (figure 5B, C) and mouse pancreatic cancer cells (figure 5D). To further test the role of Gas6 in cancer cell regrowth in metastatic livers after gemcitabine treatment in humans, we generated precision cut liver slices (PCLS) from fresh liver biopsies from treatment naïve metastatic PDAC patients. Next, PCLS were treated ex vivo with gemcitabine for 24 hours, washed, and further cultured in the presence or absence of recombinant Gas6 (figure 5E). PCLS were embedded and we assessed the presence of metastatic cancer cells (Muc1^+^) and proliferating cells (Ki67^+^). In line with our previous colony formation experiments, we found an increase in proliferating metastatic cancer cells (MUC1^+^Ki67^+^) in gemcitabine treated PCLS cultures supplemented with recombinant Gas6 compared with gemcitabine treated PCLS cultures lacking recombinant Gas6 (figure 5F, G; online supplemental figure S6E, F). These experiments suggest that Gas6 is sufficient to promote the regrowth of gemcitabine-treated pancreatic cancer cells not only in PDAC cell lines in vitro, but also in patient-derived metastatic liver samples ex vivo. Taken together, these findings show that (1) the infiltration of neutrophils in metastatic lesions after chemotherapeutic treatment leads to metastatic relapse in vivo, (2) Gas6 expression is highly upregulated in neutrophils after chemotherapy and (3) Gas6 promotes regrowth of gemcitabine-treated pancreatic cancer cells.

### Blockade of Gas6/Axl signalling axis restrains metastatic relapse after gemcitabine treatment

**Figure 6:**
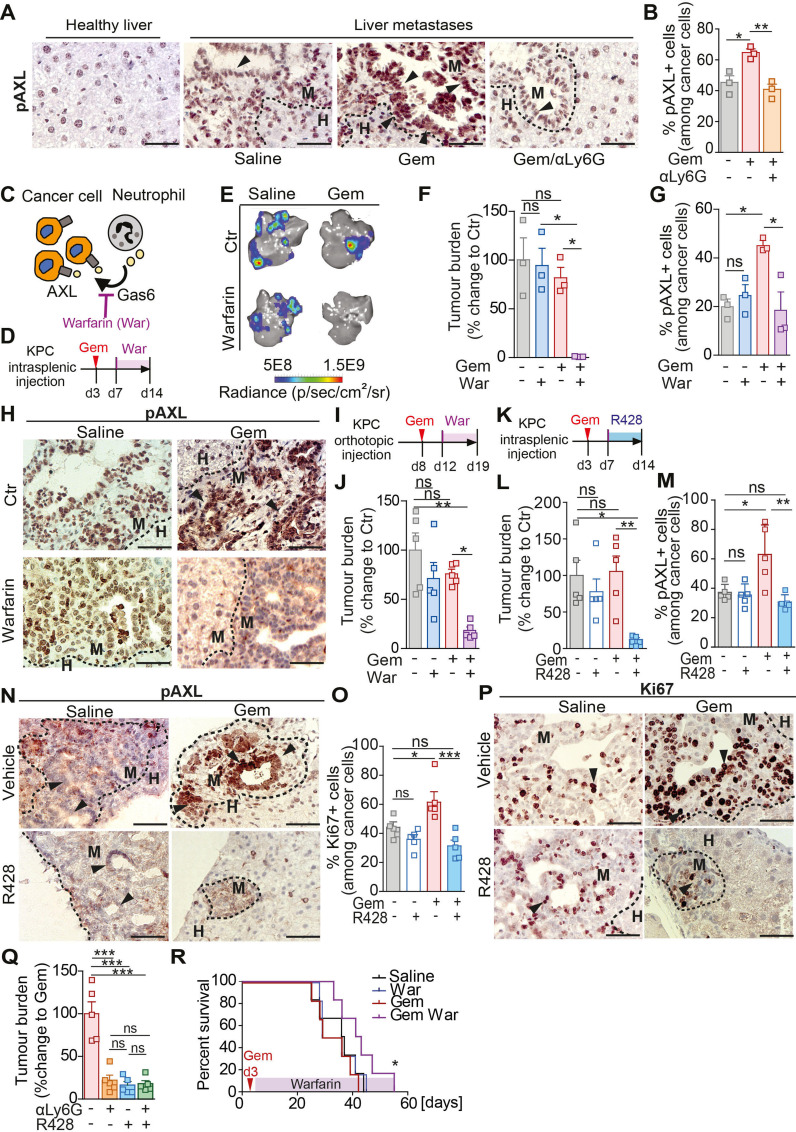
Blockade of the Gas6/Axl signalling pathway via warfarin inhibits metastatic relapse after gemcitabine treatment. (A–B) Representative images of pAXL staining in liver tissue sections derived from naïve mice or metastasis bearing mice treated with saline (control) or treated with gemcitabine alone (GEM) or GEM + αLy6G (A). Quantification of pAXL+ tumour cell frequency (B) (n=3 mice/group). (C) Schematic illustrating Gas6/Axl blockade via warfarin. (D–H) Liver metastasis was induced by intrasplenic implantation of KPC^luc^/^zsGreen^ cells. At day 3, mice were treated with gemcitabine (GEM) or saline control (saline), at day 7 mice were treated with warfarin (war) or left untreated (CTR). (D) Schematic illustration of the experiment. (E, F) Representative images of BLI signal detected in tumour-bearing livers ex vivo (E) and quantification of tumour burden by ex vivo bioluminescence imaging (BLI) (F) (n=3 mice/group). (G, H) Quantification of pAXL+ tumour cell frequency (G) and representative images of pAXL staining of metastatic tumour lesions (H). Arrowheads indicate metastatic cancer cells staining positive for pAXL (n=3 mice/group). (I, J) Primary tumour burden was induced by orthotopic implantation of KPC^luc^/^zsGreen^ cells into the pancreas. At day 8, cohorts were treated with GEM or saline control. Treatment with warfarin started at day 12. Livers were resected at day 19 and assessed for metastatic tumour burden (n=5 mice/group). (I) Schematic illustration of the experiment. (J) Quantification of tumour burden by ex vivo BLI (n=5 mice/group). (K–P) Liver metastasis was induced by intrasplenic implantation of KPC^luc^/^zsGreen^ cells. At day 3, mice were treated with GEM or saline, at day 7 mice were treated with R428 or control vehicle. (K) Schematic illustration of experiment. (L)Change in tumour burden was quantified by ex vivo BLI (n=4 mice/group). (M, N) Quantification of pAXL+ tumour cell frequency (M) and representative images (N). (O, P) Quantification of Ki67+ tumour cell frequency (O) and representative images (P). Arrowheads indicate metastatic cancer cells staining positive for pAXL (N) or Ki67 (P). (Q) Liver metastasis was induced by intrasplenic implantation of KPC^luc^/^zsGreen^ cells. At day 3, mice were treated with GEM, at day 4, mice were treated with αLy6G, at day 7 mice were treated with R428 or control vehicle until end point (day 14). Quantification of tumour burden by ex vivo BLI imaging (n=5 mice/group). (R) Liver metastasis was induced by intrasplenic implantation of 1×10^6^ KPC^luc^/^zsGreen^ cells. At day 3, animals were treated with GEM or saline control (saline). From day 7, mice were treated with warfarin or left untreated. Survival analysis of gemcitabine, gemcitabine/warfarin, warfarin and saline treated mice-bearing liver metastasis; log-rank (Mantel-Cox) test, p=0.0456 (GEM vs Gem/War). Median survival for saline was 36.5 days (n=6 mice), for warfarin 32.5 days (n=6 mice), gemcitabine 32.5 days (n=6 mice) and gemcitabine/warfarin 42 days (n=6 mice). Scale bar 50 µM. Data are presented as mean±SEM. Unpaired t-test or ANOVA with Bonferroni was used to calculate p values. *P<0.05; **p<0.01; ***p<0.001. ANOVA; analysis of variance; H, healthy liver; M, metastases; n.s., not significant.

Gas6 is a ligand of the TAM receptor family (Tyro3, AXL, Mer) and binding of Gas6 to a TAM receptor results in its phosphorylation and activation.^32^ The TAM receptor AXL is highly expressed by pancreatic cancer cells and its activation is associated with poor prognosis.^28^ We hypothesised that Gas6 expressing neutrophils activates AXL on disseminated pancreatic cancer cells, thereby enabling cancer cell regrowth. To test this, we assessed AXL phosphorylation in metastatic lesions of mice treated with gemcitabine or saline and in the presence or absence of neutrophils. In agreement with our hypothesis, we found that in metastatic lesions derived from gemcitabine treated mice the level of AXL phosphorylation (pAXL) in disseminated cancer cells was markedly increased compared with control (saline treated) metastatic tumour lesions (figure 6A). Importantly, depletion of neutrophils (αLy6G) abolished AXL activation on disseminated cancer cells (figure 6A, B). These data confirm that AXL receptor activation on disseminated cancer cells requires the presence of neutrophils. We next tested whether pharmacological blockade of the Gas6/AXL signalling pathway using warfarin provides a therapeutic benefit when combined with chemotherapy. Gas6 belongs structurally to the family of plasma vitamin K-dependent proteins and its biological function is dependent on ϒ-carboxylation, a process that can be blocked using warfarin^33 34^ (figure 6C). Liver metastasis was induced by intrasplenic implantation of KPC cells. After 3 days, mice-bearing micrometastatic lesions were treated with gemcitabine or saline (control) and, 4 days later, we started the treatment with warfarin (figure 6D) to avoid interference with the initial anti-tumour immune response previously observed (figure 2). Gemcitabine or warfarin as monotherapies did not affect hepatic metastatic tumour burden. However, gemcitabine treatment followed by subsequent warfarin administration markedly reduced regrowth of metastatic lesions (figure 6E, F). As expected, warfarin treatment abolished the previously observed increase in AXL activation (pAXL) in cancer cells in the gemcitabine treated cohort (figure 6G, H) and cancer cell proliferation (Ki67^+^) rates were reduced (online supplemental figure S7A, B), while neutrophil numbers remained unchanged (online supplemental figure S7C, D). We and others previously showed that Gas6/AXL signalling also inhibits NK cell activation and warfarin treatment increases NK cell activation and reduces pulmonary metastasis in pancreatic cancer.^35 36^ Thus, we assessed the infiltration of NK cells in hepatic metastatic lesions using the NK activation marker NKp46. We found that NK cell numbers in the hepatic metastatic lesions were very low and not affected by warfarin (online supplemental figure S7E, F).

We next assessed whether warfarin is capable of inhibiting metastatic growth of disseminated cancer cells in a spontaneous metastasis model of pancreatic cancer after chemotherapy treatment. To test this, KPC derived cells were orthotopically implanted into the pancreas. At day 8, after the establishment of primary tumours, animals were treated with gemcitabine, followed by warfarin administration (figure 6I). Gemcitabine and warfarin administration alone, or as combinatorial therapy, did not affect primary tumour burden (online supplemental figure S7G, H). Similar to the experimental metastasis model, gemcitabine and warfarin treatment as monotherapies did not affect metastatic tumour burden compared with control treated mice. However, the presence of warfarin following gemcitabine treatment significantly reduced metastatic tumour burden (figure 6J), AXL activation (pAXL) and proliferation (Ki67^+^) of disseminated cancer cells (online supplemental figure S7I–L), but immune cell infiltration remained unchanged by warfarin (online supplemental figure S7M–Q). To further confirm that Gas6 mediates metastatic relapse through AXL activation and not through one of its other TAM receptors (Tyro3, Mer), we next tested the effect of the AXL inhibitor R428 on metastatic relapse after treatment (figure 6K). In line with our previous findings, pharmacological inhibition of AXL after gemcitabine withdrawal significantly reduced metastatic relapse (figure 6L; online supplemental figure S8A, B), AXL (pAXL) activation and proliferation (Ki67+) of metastatic cancer cells (figure 6M–P), while immune cell infiltration remained unaffected (online supplemental figure S8C–G). Consistent with our findings that metastasis associated neutrophils are a main source of Gas6 expression and are critical for the activation of AXL after chemotherapy withdrawal, AXL inhibition in neutrophil depleted mice did not show any additional therapeutic benefit compared with neutrophil depletion alone (figure 6Q). Moreover, pharmacological inhibition of the Gas6/AXL signalling pathway with warfarin after gemcitabine treatment significantly increased overall survival of mice with liver metastases (figure 6R).

Thus, our findings provide evidence that targeting the AXL receptor is sufficient to reduce metastatic relapse after gemcitabine treatment.

### Chemotherapy treatment upregulates the expression of *Cxcl1* and *2* in pancreatic cancer cells which promotes neutrophil recruitment to the tumour site

**Figure 7:**
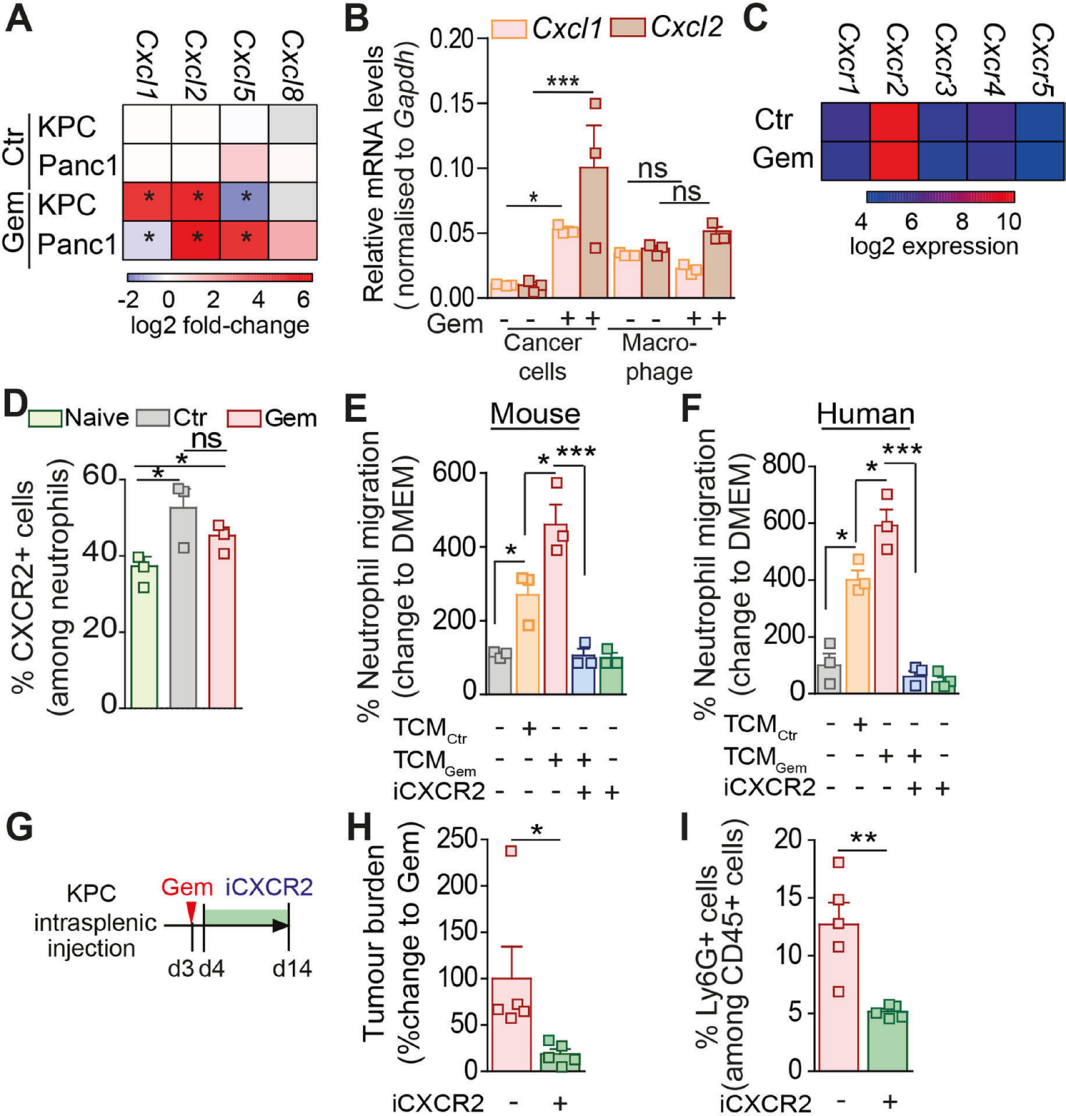
Chemotherapy treatment upregulates the expression of the neutrophil chemo-attractants CXCL1 and 2 in disseminated tumour cells. (A) Heatmap depicting *Cxcl1, 2, 5, 8* mRNA expression levels assessed by real time PCR in KPC (murine) and PANC-1 (human) pancreatic cancer cell lines untreated (CTR) and gemcitabine treated (GEM) (three independent experiments; mean±SEM). (B–D) Liver metastasis was induced by intrasplenic implantation of KPC cells. Cohorts were treated at day three with saline (CTR) or GEM (n=3 mice/group). Cancer cells, macrophages and neutrophils were isolated from metastatic lesions at day 14 by FACS. (B) Quantification of *Cxcl1* and *2* mRNA levels by real time PCR in disseminated KPC cancer cells and macrophages (three independent experiments; mean±SEM). (C) Heatmap depicting gene expression analysis of CXCR family receptors (*Cxcr1,2, 3, 4*) by metastasis infiltrating neutrophils. (D) Quantification of CXCR2 expression levels by flow cytometry on neutrophils isolated from tumour-free livers (naïve) and liver metastases derived from saline (CTR) or gemcitabine (GEM) treated mice (n=3 mice/group). (E, F) Quantification of murine (E) and human (F) neutrophil migration in the presence and absence of CXCR2 inhibitor SB225002 (iCXCR2) towards tumour conditioned media (TCM) generated from pancreatic cancer cells (KPC and Panc1, respectively) exposed to gemcitabine (TCM_Gem_) or control (TCM_Ctr_) (three independent experiments; mean±SEM). (G–I) Liver metastasis was induced by intrasplenic implantation of KPC cells. Cohorts were treated at day three with GEM or saline (CTR). From day 4 mice were treated with SB225002 (iCXCR2) until endpoint (day 14). (H) Quantification of tumour burden by ex vivo BLI (n=5 mice/group). (I) Flow cytometry quantification of neutrophil frequency in metastatic livers at endpoint. Data are presented as mean±SEM. Unpaired t-test or ANOVA with Bonferroni was used to calculate p values. *P<0.05; **p<0.01; ***p<0.001; ANOVA, analysis of variance; n.s., not significant.

Chemotherapies often show adverse side effects in patients, including a transient reduction of immune cell populations caused by the toxicity of the drugs.^37^ Hence, we further analysed neutrophil numbers in the peripheral blood of liver metastasis bearing patients and mice in response to chemotherapy treatment. As expected, a single dose of chemotherapeutic agents was sufficient to induce a transient reduction in neutrophil numbers in the blood of patients and mice, while 2 weeks after the last dose of treatment, neutrophil numbers rebounded (online supplemental table S3; online supplemental figure S9A, B). To explore the mechanism promoting the accumulation of neutrophils at the metastatic site in response to chemotherapy after rebound, we next tested whether pancreatic cancer cells treated with chemotherapeutic drugs upregulate the expression of chemokines known to promote neutrophil migration, including CXCL1, 2, 5 and 8 (expressed in human only).^38^ We found that in human and mouse pancreatic cancer cells, the chemokines *Cxcl1* and *Cxcl2* are highly upregulated in response to gemcitabine treatment (figure 7A). The upregulation of *Cxcl1,2* by cancer cells in response to gemcitabine treatment was further confirmed in flow cytometry sorted disseminated pancreatic cancer cells (figure 7B). Interestingly, we also found that within the metastatic site, macrophages are a substantial source of *Cxcl1* and *2* expression but their expression levels remained unaffected in response to gemcitabine (figure 7B). Since CXCL1,2,5 and 8 bind to the chemokine receptor CXCR2 which is associated with neutrophil recruitment to tumours,^8^ we first confirmed that *Cxcr2* is indeed highly expressed in metastasis infiltrating neutrophils (figure 7C) and that CXCR2 expression is not affected by gemcitabine treatment (figure 7D; online supplemental figure S9C). Next, we tested whether pharmacological blockade of CXCR2 was sufficient to ablate neutrophil migration. As expected, while tumour conditioned media generated from gemcitabine-treated human and mouse pancreatic cancer cells significantly increased neutrophil migration in vitro, compared with control conditioned media, the presence of the CXCR2 inhibitor SB225002 inhibited neutrophil migration (figure 7E, F; online supplemental figure S9D, E). Moreover, recombinant CXCL2 was sufficient to induce human and mouse neutrophil migration (online supplemental figure S9F, G). In vivo, pharmacological inhibition of CXCR2 (figure 7G) significantly inhibited metastatic relapse after gemcitabine withdrawal (figure 7H; online supplemental figure S9H) and reduced the accumulation of neutrophils at the metastatic site (figure 7I; online supplemental figure S9I). Taken together, these results show that the neutrophil attracting cytokines *Cxcl1* and *2* are highly expressed in metastatic livers in response to gemcitabine withdrawal and this favours CXCR2-dependent recruitment of neutrophils at the hepatic metastatic site.

10.1136/gutjnl-2021-325272.supp4 Supplementary data

### Chemotherapy treatment induces accumulation of Gas6 expressing neutrophils in liver metastases of patients with stage IV colorectal cancer

**Figure 8:**
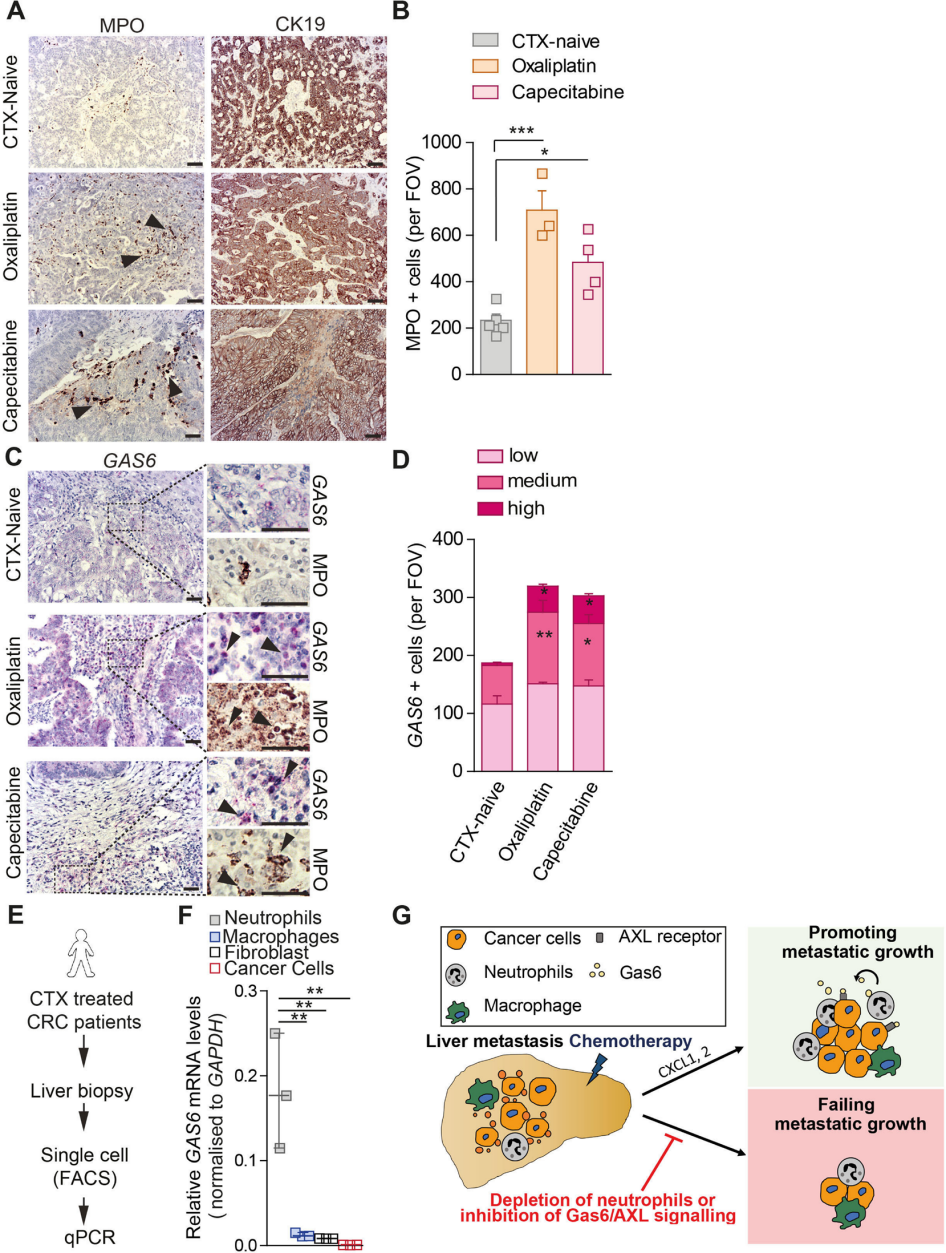
Chemotherapy treatment induces accumulation of Gas6 expressing neutrophils in liver metastases of patients with stage IV colorectal cancer. (A–D) Tissue sections from metastatic livers derived from treatment naïve patients with stage IV colorectal cancer (CRC) (n=5), and patients undergone treatment with oxaliplatin (n=3) or capecitabine (n=4) were stained for cancer cells (CK19), neutrophils (MPO) and *GAS6*. (A, B) Representative images of CK19 and MPO staining of serial sections (A) and quantification of data (B). (C, D) Representative images of *GAS6* and MPO staining of serial sections (C) and quantification of data (D). Arrowheads indicate *GAS6+* staining in neutrophil-rich areas. (E, F) Liver biopsies were collected from metastatic CRC patients post-FOLFOX treatment. Cell populations were isolate by FACS for gene expression analysis. (G) Schematic illustration of experiment and (H) quantification of *GAS6* mRNA levels by real-time PCR in neutrophils, macrophages, fibroblast and cancer cells (n=3 patient samples). (G) Schematic depicting the role of neutrophil-derived Gas6 in hepatic metastatic tumour regrowth after chemotherapy in pancreatic cancer. Chemotherapy induces the expression of the neutrophil-chemoattractants CXCL1 and 2 by disseminated cancer cells. On treatment withdrawal, neutrophils are recruited to the liver and express high levels of Gas6. Neutrophil-derived Gas6 activates AXL receptors on disseminated cancer cells and promotes their regrowth after chemotherapeutic treatments. Depletion of neutrophils or inhibition of Gas6/AXL signalling axis inhibits metastatic regrowth of pancreatic cancer cells. Scale bar 50 µM. Data are presented as mean±SEM. Unpaired t-test or ANOVA with Bonferroni was used to calculate p values. *P<0.05; **p<0.01; ***p<0.001. ANOVA, analysis of variance; H, healthy liver; M, metastases; n.s., not significant.

Like pancreatic cancer, colorectal cancer (CRC) frequently metastasises to the liver.^39^ While in pancreatic cancer, liver biopsies are only taken prior chemotherapeutic intervention for diagnostic purpose,^25^ in CRC, chemotherapy is often the standard-of-care treatment for patients with stage IV CRC (patients with liver metastasis), prior to their metastatic liver surgical resection.^40^ These differences in patient care provided us an opportunity to collect liver samples from patients with stage IV CRC treated with chemotherapy, and to analyse whether metastatic liver tumours from patients with CRC show an increase in neutrophil infiltration and Gas6 expression in response to chemotherapy. We analysed resected metastatic liver tumours from untreated patients with stage IV CRC, and from patients with stage IV CRC treated with capecitabine or oxaliplatin. We found that metastatic tumour cells (cytokeratin 19^+^) in both chemotherapy-treated patient cohorts were surrounded by higher numbers of neutrophils (MPO^+^) compared with the untreated patient cohort (figure 8A, B). Analysis of serial tissue sections revealed increased levels of Gas6 +expression in neutrophil-rich areas in the treated patients (figure 8C, D; online supplemental figure S9J). To further confirm that neutrophils are indeed a major source of Gas6 expression in human liver metastases after chemotherapy treatment, we enzymatically disaggregated fresh liver samples from chemotherapy-treated patients with CRC into single cell suspensions and isolated neutrophils, macrophages, fibroblasts and cancer cells by flow cytometry-based cell sorting (figure 8E; online supplemental figure S9K). Subsequent gene expression analysis confirmed that neutrophils are the cells expressing the highest levels of *GAS6* in patient liver metastases after chemotherapy cessation (figure 8F). Taken together, these findings suggest that chemotherapy-induced neutrophil accumulation and upregulation of Gas6 also occurs in liver metastasis of patients with CRC and thus, targeting Gas6 might improve therapeutic interventions in patients with pancreatic cancer and CRC.

## Discussion

Our findings demonstrate that standard cytotoxic chemotherapy temporarily restrains metastatic PDAC progression but, also induces cellular changes in the metastatic tumour microenvironment which subsequently promote metastatic relapse. Specifically, we found that chemotherapy induces the expression of neutrophil chemoattractants in tumour cells and subsequent recruitment and infiltration of Gas6 expressing neutrophils to the liver in a CXCR2-dependent manner. Neutrophil-derived Gas6 then activates the receptor tyrosine kinase AXL on metastatic cancer cells and promotes metastatic growth in the liver (figure 8G). In this study, we also show that pharmacological inhibition of Gas6/AXL signalling in combination with chemotherapy ablates metastatic relapse, thereby providing the rationale for further evaluating this therapeutic strategy for PDAC patients.

Primary pancreatic tumours are largely refractory to chemotherapeutic treatments and surgical resection remains the only curative treatment option.^25^ Reasons for this include the excessive stromal compartment, hypovascularisation and increased interstitial fluid pressure within PDAC tumours, all acting as a barrier for efficient drug delivery.^41^ Patients that have undergone surgery also receive adjuvant chemotherapy as standard of care.^25^ In a preclinical mouse model of PDAC, adjuvant gemcitabine treatment, after the resection of the pancreatic tumour, inhibited local recurrence at the primary tumour site, but not at the distant metastatic sites.^42^ However, the effect of chemotherapy on metastatic tumours, and micrometastases in the adjuvant setting, remains poorly understood. Our results provide evidence that metastatic PDAC lesions are responsive to chemotherapeutic treatment, particularly during early metastatic development where the stromal compartment is less established.^3^ In fact, chemotherapy increases cancer cell death and the release of tumour antigens, a critical step in the generation of an antitumour immune response.^43^ In agreement with this, we observe in our PDAC metastatic mouse model that chemotherapy induces an initial proinflammatory response with activation of cytotoxic T cells in the metastatic liver niche. Although this proinflammatory response was only transient, it might provide a window of opportunity for the use of immune checkpoint inhibitors in combination with chemotherapy to further boost this initial T cell activation. Similar to what we observed in the adjuvant setting at the hepatic metastatic site in murine models, in the neoadjuvant setting for PDAC patients, chemotherapy has been shown to restore an anti-tumour immune response at the primary site associated with a decrease in immune suppressive myeloid cells, T_regs_, and CD163^+^ macrophages.^44 45^ Immune suppressive myeloid Ly6G^+^ cells suppress CD8^+^ T cell functions in primary PDAC tumours.^8 46^ Our findings suggest that in liver metastases the presence of immune suppressive M2-like macrophages is key to inhibit the initial chemotherapy-induced antitumour immune response. In contrast, neutrophils are dispensable for the maintenance of an immunosuppressive metastatic tumour microenvironment. This might be due to the fact that macrophages are present in livers in a much higher number compared with neutrophils.^3^ We and others have previously shown that metastasis associated macrophages can suppress CD8^+^ T cell functions in the liver.^12 13^ In agreement with these findings, the use of a CSF-1/CSF-1R inhibitors also reduced metastatic relapse after gemcitabine treatment, confirming that macrophages play a key role in controlling the local immune response in liver metastasis. Since macrophages have a high plasticity and their heterogeneity is diverse in the liver, future treatments should focus on targeting macrophage immune-suppressive functions or on inhibiting macrophage polarisation towards an immunosuppressive phenotype. Neutrophils have been associated with different prometastatic functions, mainly at the early steps of the metastatic cascade or even at the premetastatic niche formation.^8 9 18 47^ Here, we have identified a novel prometastatic function of neutrophils in promoting metastatic growth of cancer cells after the initial colonisation steps. Mechanistically, we found that cessation of chemotherapy induces the infiltration of neutrophils and that neutrophils activate AXL receptor on metastatic cancer cells, via secretion of the AXL receptor ligand Gas6, leading to cancer cell growth at the metastatic liver. AXL is overexpressed in pancreatic cancer and is associated with increased metastasis and a poor prognosis.^28^ The Gas6/AXL signalling pathway regulates several cancer cell autonomous and non-cancer cell autonomous processes.^48 49^ AXL has been found to induce epithelial-mesenchymal transition in pancreatic cancer cells and thereby promote cancer cell migration, invasion, and metastatic spreading in vivo. Moreover, genetic depletion of AXL in pancreatic cancer cells increased their sensitivity to chemotherapy.^31 33 50^ In our studies, we started AXL inhibition after the initial seeding and colonisation step of the liver and after the exposure to gemcitabine treatment. Hence, our data reveal an additional novel role of AXL signalling in promoting the regrowth of cancer cells after chemotherapy in established distant lesions, thereby expanding the potential use of AXL inhibitors to fight pancreatic cancer. We and others have previously shown that Gas6 is also expressed by macrophages and fibroblasts^51^ and that inhibition of Gas6/AXL signalling increases NK cell activation and reduces pulmonary metastasis.^35 36^ Interestingly, here, we show that at the hepatic metastatic site, basal Gas6 expression levels are very low, but are markedly increased in the liver-stroma of human and mouse in response to chemotherapeutic treatments, and are mainly expressed by the infiltrated neutrophils. Moreover, we found that hepatic metastatic lesions are poorly infiltrated by NK cells and neither their numbers nor their activation status was altered by warfarin or AXL inhibition, suggesting that the multifunctional Gas6/AXL signalling pathway regulates different processes and cell populations in different organs, thereby contributing in many different ways to PDAC metastasis.

Our studies exemplify two therapeutic options to inhibit metastatic relapse by using warfarin or the AXL inhibitor R428 (Bemcentinib). Both agents are currently tested in patients with pancreatic cancer (NCT03536208) and (NCT03649321), respectively. Hence, our findings further strengthen the rationale for targeting Gas6/AXL signalling in the treatment of metastatic PDAC and, in combination with chemotherapy, to reduce the risk of recurrence in the adjuvant setting by preventing the progression of micrometastatic disease. Further studies will be needed to explore the mechanism by which chemotherapy withdrawal induces Gas6 expression in neutrophils, and to test whether Gas6 levels in circulating neutrophils could be also used as a biomarker for predicting the risk of metastatic recurrence in the adjuvant setting.

Our additional analysis of liver metastases from patients with stage IV CRC further suggests that the observed increase in Gas6-expressing neutrophils in response to chemotherapy might not be restricted to pancreatic cancer metastasis, but may also occur in other cancers that metastasise to the liver. Thus, targeting the identified neutrophil/Gas6/AXL axis might also be of relevance for cancers with a high prevalence to spread to the liver, such as CRC, melanoma, breast and lung.^39^ In summary, our findings are important and timely as they could help improve in the near future the design of treatment regimens for patients with cancer with liver metastases.

## Materials and methods

Detailed materials and methods can be found in online supplemental section.

## Data Availability

All data relevant to the study are included in the article or uploaded as online supplemental information.
