# Supplementary material for: Chemotherapy-induced infiltration of neutrophils promotes pancreatic cancer metastasis via Gas6/AXL signalling axis
Source: Gut. 2022 Jan 12;71(11):2284–99. doi: 10.1136/gutjnl-2021-325272 (PMC9554050; doi:10.1136/gutjnl-2021-325272)
Supplement: Supplementary data [file gutjnl-2021-325272supp001.pdf]

SUPPLEMENTAL MATERIAL

Chemotherapy-induced infiltration of neutrophils promotes pancreatic cancer metastasis via Gas6/AXL signalling axis.

AUTHORS:

Gaia Bellomo[1#], Carolyn Rainer[1#], Valeria Quaranta[1], Yuliana Astuti[1], Meirion Raymant[1], Elzbieta Boyd[1], Ruth Stafferton[1], Fiona Campbell[1], Paula Ghaneh[1], Christopher Halloran[1], Dean E Hammond[2], Jennifer P Morton[3], Daniel Palmer[1], Dale Vimalachandran[1], Robert Jones[1], Ainhoa Mielgo[1] & Michael C Schmid[1*]

[1] Department of Molecular and Clinical Cancer Medicine, University of Liverpool, Ashton Street, Liverpool, L69 3GE, UK.

[2] Department of Molecular Physiology and Cell Signalling, University of Liverpool, Ashton Street, Liverpool, L69 3GE, UK.

[3]Cancer Research-UK Beatson Institute and Insitute of Cancer Sciences, University of Glasgow, Switchback Road, Glasgow, G61 1BD

[#]Equally contributing authors

[*] to whom correspondence should be addressed mschmid@liverpool.ac.uk

Bellomo G, *et al. Gut* 2022; 71:2284–2299. doi: 10.1136/gutjnl-2021-325272

SUPPLEMENTARY FIGURE LEGENDS

Supplementary Figure S1: Characterisation of gemcitabine induced pancreatic cancer cell death *in vitro* and *in vivo*.

(A) Quantification of caspase 3-7 activities in KPC cells untreated or treated with increasing concentrations of gemcitabine (Gem: 10 nM, 50 nM, 100 nM and 200nM) for 24h and 48h (three independent experiments, mean ± SEM). One-way ANOVA with Dunnett's multiple comparison test to 0 nM.

(B-I) Liver metastasis was induced by intrasplenic implantation of $1x10^6$ $KPC^{luc}/^{zsGreen}$ cells. Starting from day 12, animals were treated with gemcitabine (Gem; 100 mg/kg) or saline (Ctr) every 3rd day with 4 doses in total.
(B, C) Representative H&E images (B) and quantification (C) of metastatic lesion area after treatment (day 22).
(D, E) Representative H&E images (D) and quantification (E) of metastatic lesion area at human endpoint (HEP) (n= 5 mice/group/time point).
(F, G) Tumour burden assessed *ex vivo* by bioluminescence imaging (BLI) at humane endpoint (HEP) in spleen and liver (n= 14 mice/group) (F) and representative BLI images (G).
(H, I) Representative IHC images (H) and quantification (I) of apoptotic KPC cells staining positive for cleaved caspase 3 (CC3) at HEP (n= 5 mice /group). Arrowheads indicate apoptotic ($CC3^+$) cancer cells in ductal structures.

(J, K) Liver metastasis was induced by intrasplenic implantation of $5x10^5$ $KPC^{luc}/^{zsGreen}$ cells and animals received one dose of gemcitabine (Gem; 100 mg/kg) or saline (Ctr) at day 3. Representative immunofluorescence images (J) and quantification (K) of apoptotic $KPC^{luc}/^{zsGreen}$ cells staining positive for TUNEL at day 14 in saline (Ctr) and Gem treated mice (n= 5 mice /group). Arrowheads indicate apoptotic ($TUNEL^+$) cancer cells.

(L) Flow cytometry gating strategy for dendritic cell (DC) populations ($CD103^+$ and $CD11b^+$) and macrophages (Macs). Cells were selected by size using SSC-A vs FSC-A; dead cells were excluded using Sytox blue; live cells were gated for $CD45^+$ cells and subsequentially gated on F4/80 to separate Macs, and CD11c for total DC; dendritic cells were further separated in the $CD103^+$ and $CD11b^+$ subpopulations. Macs, $CD103^+$ and $CD11b^+$ DCs were gated on zsGreen to detect uptake of KPC cells.
Scale bars = 100 µM (B, D); scale bar 50 µM (H, J); M = metastases, H = healthy liver;
Data are presented as mean ± SEM. Unpaired t-test or ANOVA was used to calculate P values. *$P < 0.05$; $**P < 0.01$; $***P < 0.001$;* n.s., not significant.

Supplementary Figure S2: Gating strategies used for mass cytometry-based analysis of immune cell infiltrates in pancreatic liver metastases in response to chemotherapy.

(A, B) Liver metastasis was induced by intrasplenic implantation of $KPC^{luc/zsGreen}$ cells and animals were treated with gemcitabine (Gem; 100mg/kg) or saline (Ctr) at day 3. Metastatic livers were resected at initial response (day 4) and after treatment withdrawal (day 14). Unsupervised down-sampling was performed with viSNE using equal numbers of manually gated viable $CD45^+$ cells. (A) Representative viSNE maps showing individual markers highlighted in a dot plot on the axes tSNE1 and tSNE2 for gemcitabine treated samples, with each dot representing a single cell. Resulting cell clusters were used to delineate main immune cell populations. (B) Main immune cell populations as shown in (A) were further analyses for expression of surface molecules associated with an activated phenotype (CD69, CD86, MHC class II) (Ctr d4 n= 4 mice, Gem d4 n= 4 mice; Ctr d14 n= 3 mice; Gem d14 n= 4 mice).

Supplementary Figure S3: $CD4^+$ T cell numbers are increased during the initial response to gemcitabine, while CD206+ and YM1+ macrophage numbers are reduced.

(A-L) Liver metastasis was induced by intrasplenic implantation of $KPC^{luc/zsGreen}$ cells and animals were treated with gemcitabine (Gem; 100mg/kg) or saline (Ctr) at day 3. Metastatic livers were resected at initial response (day 4) and after treatment withdrawal (day 14) (n= 5 mice/group/time point A-F; n= 3 mice/group/time point G-L).

(A) Flow cytometry gating strategy used to assess T cell subpopulations ($CD8^+$, $CD4^+$ and $FoxP^+$ T_{reg} cells) and (B-D) quantification of data.

(E) Flow cytometry gating strategy used for active ($CD69^+$) $CD4^+$ T cells and (F) quantification of data (n= 5 mice /group).

(G, H) Flow cytometry gating strategy applied (G) and quantification (H) of $CD206^+$ cells among macrophages ($F4/80^+$ cells) in metastatic livers of Gem or saline treated animals during initial response (n=3 mice/group).
(I, J) Representative immunohistochemistry images (I) and quantification (J) of YM-1 positive cells in livers of Gem or saline treated mice during initial response (n=3 mice/group).
(K, L) Representative immunohistochemistry images (K) and quantification (L) of $YM\text{-}1^+$ cells in livers of Gem or saline treated mice after withdrawal (n=3 mice/group).

Scale bar 50 µM; M = metastases, H = healthy liver; Arrowheads indicate YM-1$^+$ cells (macrophages). Data are presented as mean ± SEM. Unpaired t-test was used to calculate P values **P < 0.05; ***P < 0.001;* n.s., not significant.

Supplemental Figure S4: Macrophage-targeted therapy reduces PDAC liver metastasis independent of gemcitabine treatment, while depletion of neutrophils reduced metastatic tumour burden only after gemcitabine withdrawal.

(A-C, F-H) Liver metastasis was induced by intrasplenic implantation of KPC^{luc}/^{zsGreen} cells and animals were treated with gemcitabine (Gem; 100mg/kg) or saline (Ctr) at day 3. At day 4, mice were treated with IgG control or αLy6G antibody for 2 weeks. (A) Flow cytometry quantification of neutrophils (CD45$^+$CD11b$^+$Ly6G$^+$Ly6C^{low} cells) frequency among immune cells (CD45$^+$ cells) in peripheral blood of Ctr + IgG treated, Ctr + αLy6G antibody treated, Gem + IgG treated, and Gem + αLy6G antibody treated mice at endpoint (day18) (n= 3 mice/group). (B-C) Tumour burden quantification by BLI of gemcitabine treated mice (B) receiving isotope control antibody (Gem + IgG) or monoclonal antibody targeting neutrophils (Gem + αLy6G) (n= 3 mice /group) and saline treated mice (C) receiving isotope control antibody (Ctr + IgG) or monoclonal antibody targeting neutrophils (Ctr + αLy6G) (n= 3 mice /group).

(D-H, M, N) Liver metastasis was induced by intrasplenic implantation of KPC^{luc}/^{zsGreen} cells and animals were treated with gemcitabine (Gem; 100mg/kg) or saline (Ctr) at day 3. At day 4, mice were treated with IgG control or αCSF1 antibody for two weeks. Tumour burden quantification by BLI of gemcitabine treated mice (D) receiving isotope control antibody (Gem + IgG) (n= 3 mice /group) or monoclonal antibody targeting macrophages (Gem + αCSF-1) (n= 4 mice /group) and saline treated mice (E) receiving isotope control antibody (Ctr + IgG) or monoclonal antibody targeting macrophages (Ctr + αCSF1) (n= 3 mice /group).

(F-H) At endpoint (d18), liver tumours were surgically resected and analysed by flow cytometry for macrophage and neutrophil infiltration. (F) Flow cytometry gating strategy and (G) quantification of macrophages in response to neutrophil-depletion (αLy6G) and macrophage-depletion (αCSF-1) and (H) neutrophil numbers in response to neutrophils-depletion (αLy6G) and macrophage-depletion (αCSF-1).

(I-J) Liver metastasis was induced by intrasplenic implantation of KPC^{luc}/^{zsGreen} cells and animals were treated with gemcitabine (Gem; 100mg/kg) at day 3. At day 4, mice were treated with αLy6G or IgG controls for 2 weeks; at day 7, mice were treated with αCD8 or

IgG controls until endpoint (day 14). Flow cytometry quantification of neutrophil ($CD45^+CD11b^+Ly6G^+Ly6C^{low}$ cells) frequency among immune cells ($CD45^+$ cells) in peripheral blood of Ctr + IgG treated, Ctr + αLy6G, Ctr + αLy6G and αCD8 antibody treated (I), and of CD8 T cell ($CD45^+CD3^+CD8^+$ cells) frequency among immune cells ($CD45^+$ cells) in peripheral blood of Ctr+ IgG treated, Ctr + αLy6G, Ctr + αLy6G and αCD8 antibody treated (n= 5 mice /group).

(K) Colony formation assay of KPC cells gemcitabine-treated (Gem) or untreated (Ctr). Bar graph shows fold reduction of BLI signal compared to untreated (Ctr) KPC cells (three independent experiments; mean ± SEM).

(L) Quantification of colony formation assay of gemcitabine treated $KPC^{luc/zsGreen}$ cells in the presence or absence of Gas6 neutralizing antibody (anti-Gas6) with or without metastasis infiltrating macrophages ($F4/80_{Gem}$) isolated from mice treated with gemcitabine. Bar graph shows fold upregulation of BLI signal compared to gemcitabine-treated $KPC^{luc/zsGreen}$ cells alone (three independent experiments; mean ± SEM).

(M, N) Representative IHC images (M) and quantification of proliferating Ki67+ tumour cell frequency in metastatic livers (N).

(O) RNA Sequencing of metastasis infiltrating neutrophils isolated by FACS from tumour bearing livers of mice at day 14 after treatment with Gem or untreated (Ctr). Schematic illustration of experiment.

Scale bar 100 μM; M = metastases, H = healthy liver; Data are presented as mean ± SEM. s. Unpaired t-test or ANOVA with Bonferroni was used to calculate P values. *$P < 0.05$; $**P < 0.01$; $***P < 0.001$;* n.s., not significant.

Supplementary Figure S5: Standard of care chemotherapeutic treatment regimens induce the infiltration of $Gas6^+$ neutrophils into metastatic tumours.

(A) Flow cytometry gating strategy utilised for isolation of $KPC^{zsGreen}$ cancer cells, stroma cells, neutrophils and macrophages by FACS.

(B) Representative images of myeloperoxidase (MPO) and *Gas6* staining using RNAscope in metastatic tissue serial sections derived from saline (Ctr) or gemcitabine (Gem) treated mice. Arrowheads indicate *$Gas6^+$* staining in neutrophil-rich areas. Micrographs are a higher magnification of same images shown in main Figure 4F.

(C) Spontaneous liver metastases from the autochthonous mouse pancreatic cancer model *$Kras^{G12D}$;$Trp53^{R172H}$;Pdx1*-Cre (KPC mice) treated with gemcitabine (KPC Gem) or left untreated (KPC Ctr) were isolated and analysed. Representative images of MPO and *Gas6*

staining in metastatic tissue sections. Arrowheads indicate *Gas6*+ staining in neutrophil-rich areas. Micrographs are a higher magnification of the same images as shown in main Figure 4I.

(D) Representative images of *low*, *medium* and *high Gas6* expression levels in metastatic bearing liver tissue sections. Intensity threshold parameters: *low* ≤ 0.27, *medium* = 0.57-0.77, *high* ≥ 0.78.

(E, F) Liver metastasis was induced by intrasplenic implantation of $KPC^{luc}/^{zsGreen}$ cells. Mice were treated with gemcitabine or saline 3 days post cell implantation. Treatment with αLy6G or control IgG started at day 4 (n=4 mice/group). Livers were resected after 14 days. Representative images (E) and quantification (F) of *Gas6* staining using RNAscope in metastatic tissue sections from different cohorts after treatment withdrawal. Arrowheads indicate increased $Gas6^+$ staining in metastatic lesions.

(G) Flow cytometry-based quantification of neutrophils among $CD45^+$ cells in metastatic livers and tumour-free lungs of gemcitabine treated animals compared to saline (Ctr) treated animals during initial response (n=3 mice/group).

(H) Bar graph shows fold induction of *Gas6* expression in neutrophils isolated from metastatic livers or tumour-free lungs derived from Gem treated mice and untreated mice (n=3 mice/group).

(I-N) Liver metastasis was induced by intrasplenic implantation of $5x10^5$ $KPC^{luc}/^{zsGreen}$ cells and animals were treated with gemcitabine + nab-paclitaxel (Gem-Nab), FOLFIRINOX (Folfx) or saline (Ctr) at day 5. Metastatic livers were resected after treatment withdrawal (day 15) (n=4 mice/group).

(J) Tumour burden was monitored at day 3, 7 and 15 by BLI *in vivo* imaging. Change in tumour burden in Gem-Nab and Folfx treated groups compared to control group.

(K) Quantification of neutrophil frequency in metastatic livers at endpoint.

(L) Quantification of *Gas6* mRNA levels by real time PCR in intra-metastatic neutrophils ($Ly6G^+$ cells) isolated by FACS from different cohorts after treatment withdrawal.

(M, N) Representative images (M) and quantification (N) of *Gas6* staining using RNAscope in metastatic tissue sections from different cohorts after treatment withdrawal. Arrowheads indicate increased $Gas6^+$ staining in metastatic lesions.

Scale bar 100 µM; M = metastases, H = healthy liver. Data are presented as mean ± SEM. Unpaired t-test or ANOVA with Bonferroni was used to calculate P values. *$^*P < 0.05$; $^{**}P < 0.01$;* n.s., not significant.

Supplementary Figure S6: Neutrophils upregulate the expression of *Gas6* after gemcitabine treatment, while apoptotic neutrophil numbers remain the same.

Bellomo G, *et al. Gut* 2022; 71:2284–2299. doi: 10.1136/gutjnl-2021-325272

(A) Peripheral neutrophils ($CD45^{+}CD11b^{+}CD14^{-}CD15^{+}CD16^{+}$) were isolated from metastatic PDAC patient during their first cycle of gemcitabine treatment using Miltenyi MACS express kit and purity was assessed by flow cytometry (99%).

(B-D) Liver metastasis was induced by intrasplenic implantation of $5x10^{5}$ $KPC^{luc}/^{zsGreen}$ cells and animals were treated with gemcitabine (Gem) or saline (Ctr) at day 3. Metastatic livers were resected after treatment withdrawal (day 14).

(B) Fold induction of *Gas6* expression in neutrophils isolated from peripheral blood (using Miltenyi kit) of gemcitabine treated mice compared to saline treated (Ctr) mice (n=3 mice/group).

(C, D) Representative immunofluorescent images (C) of apoptotic neutrophils staining positive for TUNEL at day 14 and quantification (D) of the data (n = 5 mice/group; white arrowheads indicate apoptotic neutrophils).

(E, F) Human precision cut liver slices (hPCLSs) were initially treated with gemcitabine for 24 hours then cultured in the presence or absence of rGas6 for the following 24 hours. hPCLSs were assessed by MUC1 (cancer cell marker) and Ki67 staining (proliferation marker) in serial sections. Representative images (E) and quantification (F) of proliferating $Ki67^{+}$ tumour cell frequency in *ex vivo* treated hPCLS (n=5 patient biopsies). Arrowheads indicate $Ki67^{+}$ cells in cancer cells enriched area.

Scale bar 50 µM; M = metastases, H = healthy liver. Data are presented as mean ± SEM. s. Unpaired t-test was used to calculate P values. **P < 0.05; **P < 0.01;* n.s., not significant.

Supplementary Figure S7: Combinatorial therapy using gemcitabine and warfarin inhibits metastatic relapse in experimental and spontaneous metastasis models.

(A-F) Liver metastasis was induced by intrasplenic implantation of $KPC^{luc}/^{zsGreen}$ cells. At day 3, mice were treated with gemcitabine (Gem) or saline control (Ctr), at day 7 warfarin treatment was commenced (n=3 mice/group) until endpoint (day 14).

(A, B) Representative IHC images (A) and quantification (B) of $Ki67^{+}$ cancer cells in liver tumours at endpoint.

(C, D) Representative IHC images (C) and quantification (D) of MPO^{+} neutrophils in liver tumours at endpoint.

(E, F) Representative IHC images (E) and quantification (F) of $NKp46^{+}$ NK cells in liver tumours at endpoint. Arrowheads indicate $NKp46^{+}$ NK cells.

(G-Q) Primary tumour was induced by orthotopic implantation of 2x10^5 $KPC^{luc/zsGreen}$ cells in the pancreas. At day 8, mice were treated with gemcitabine (Gem) or control saline (saline), at day 12 mice were treated with warfarin until endpoint (day19) (n=5 mice/group).

(G, H) Representative images of primary tumour (G) and quantification (H) of tumour mass at endpoint.

(I, J) Representative IHC images (I) and quantification (J) of $pAXL^+$ cancer cells in liver tumours at endpoint. Arrowheads indicate cancer cells staining positive for pAXL.
(K, L) Representative IHC images (K) and quantification (L) of proliferating $Ki67^+$ cancer cells in liver tumours at endpoint.
(M-Q) Quantification of neutrophil infiltration (M), macrophages (N, O) and NK cell (P, Q) activation at the metastatic site by flow cytometry.

Scale bar 50 µM; M = metastases, H = healthy liver. Data are presented as mean ± SEM. s. ANOVA with Bonferroni was used to calculate P values. **P < 0.05; **P < 0.01;* n.s., not significant.

Supplementary Figure S8: AXL signalling is necessary for metastatic relapse after gemcitabine treatment.

(A-G) Liver metastasis was induced by intrasplenic implantation of $KPC^{luc/zsGreen}$ cells. At day 3, mice were treated with gemcitabine (Gem) or saline (Ctr), at day 7 the treatment with R428 (AXL-inhibitor) was commenced (n=5 mice/group).

(A, B) Representative images (A) of H&E staining and quantification (B) of metastatic area in livers.

(C-E) Quantification of neutrophil infiltration (C) and macrophage activation (D, E) at the metastatic site by flow cytometry.

(F, G) Quantification of NK cell infiltration (F) and NK cell activation (G) at the metastatic site by flow cytometry.

Scale bar 50 µM; M = metastases, H = healthy liver. Data are presented as mean ± SEM. Unpaired t-test or ANOVA with Bonferroni was used to calculate P values. **P < 0.05; **P < 0.01;* n.s., not significant.

Supplementary Figure S9: Flow cytometry-based characterisation of primary neutrophils in human and mouse.

Bellomo G, *et al. Gut* 2022; 71:2284–2299. doi: 10.1136/gutjnl-2021-325272

(A) Peripheral blood was collected from metastatic PDAC patients during their first cycle of gemcitabine treatment and neutrophils absolute count [per ml] was assessed. (BL= baseline, w1 = one week after treatment, w4= four weeks after treatment) (n=2 patients).

(B) Liver metastasis was induced by intrasplenic implantation of $5x10^5$ $KPC^{luc}/^{zsGreen}$ cells and animals were treated with gemcitabine (Gem), gemcitabine + nab-paclitaxel (Gem-Nab), FOLFIRINOX (Folfx) or saline control (Ctr) at day 5. Peripheral blood was analysed by flow cytometry to assess neutrophil numbers (n=3 mice/group).

(C) Flow cytometry gating strategy to measure CXCR2 expression on murine neutrophils in metastatic liver.

(D, E) Flow cytometry analysis to assess purity of primary mouse and human neutrophils used in migration assay. (D) Purity of neutrophils isolated from murine spleen and (E) human peripheral blood.

(F) Quantification of murine neutrophil migration towards recombinant CXCL2 (rCXCL2) using migration assay (three independent experiments; mean ± SEM).

(G) Quantification of human neutrophil migration towards recombinant CXCL2 (rCXCL2) using migration assay (three independent experiments; mean ± SEM).

(H, I) Liver metastasis was induced by intrasplenic implantation of KPC cells. From day 4, mice were treated with SB225002 (iCXCR2) or saline until endpoint (day14). (H) Quantification of tumour burden by *ex vivo* BLI (n = 5 mice/group). (I) Flow cytometry quantification of neutrophil frequency in metastatic livers at endpoint.

(J) Serial sections from metastatic livers derived from treatment naïve stage IV CRC patients (n=5), and patients undergone treatment with oxaliplatin (n=3) or capecitabine (n=4) were stained for neutrophils (MPO) and *GAS6*. Micrographs are a lower magnification of the same images as shown in main Figure 8C.

(K) Flow cytometry gating strategy used for sorting immune cell populations (neutrophils, fibroblast, macrophages and cancer cells) from CRC patient liver biopsies.

Scale bar 100 µM, Data are presented as mean ± SEM. s. Unpaired t-test or ANOVA with Bonferroni was used to calculate P values. **P < 0.05; **P < 0.01;* n.s., not significant.

Bellomo G, *et al. Gut* 2022; 71:2284–2299. doi: 10.1136/gutjnl-2021-325272

SUPPLEMENTAR MATERIALS AND METHODS

Cells

The murine pancreatic cancer cells KPC FC1199 and KPC FC1245 were kindly provided by the Tuveson laboratory (Cold Spring Harbor Laboratory). KPC cells initially were isolated from PDAC tumour tissues of Kras$^{G12D/+}$; p53^{R172H}; Pdx1-Cre mice of a pure C57BL/6 background and authenticated as previously reported [1]. KPC$^{luc/zsGreen}$ cells were then generated by using pHIV Luc-zsGreen (gift from B. Welm, University of Utah, Salt Lake City, UT; Addgene plasmid no.39196) lentiviral particle infection. Infected cells were selected for high zsGreen expression levels using flow cytometry cell sorter (ARIA, BD). Human pancreatic cancer cell line PANC1 were purchased from ATCC. All cells were cultured in DMEM+10% FBS and supplemented with 2.5 µg/mL Amphotericin B (Sigma). All cells were routinely tested negative for the presence of *Mycoplasma* contamination. None of the cell lines used in this article are listed in the International Cell Line Authentication Committee and NCBI Biosample database of misidentified cell lines.

Mice

Six- to eight-week-old female C57BL/6 mice were purchased from Charles River Laboratories. All animal experiments were performed in accordance with current UK legislation under an approved project license PPL P16F36770 (M.C. Schmid). Mice were housed under specific pathogen-free conditions at the Biomedical Science Unit at the University of Liverpool. For tumour studies, female animals aged 6 to 8 weeks were used.

Metastasis studies

In the survival studies, liver experimental metastases were induced by implanting 1 x 10^6 KPC$^{luc/zsGreen}$ FC1199 in 25 µL of PBS into the spleen of immunocompetent syngeneic C57BL/6 mice using a Hamilton 29 G syringe, as previously described [2, 3]. Tumour bearing mice were treated with gemcitabine-HCl (Selleckchem, 100 mg/kg) starting from day 12 and administrated via i.p. injection in a Q3Dx4 schedule until the humane endpoint, or tumour bearing mice were treated with a single injection at day 3. For selected survival studies macrophages or neutrophils were depleted using colony-stimulating factor 1 receptor (CSF-1R) –neutralizing antibody (BioXCell, clone AFS98) or Ly6G-neutralizing antibody (BioXCell, clone 1A8), respectively. Anti-CSF1R was administered via i.p. injection with an initial dose of 400 µg and subsequent doses every other day containing 200 µg. Anti-Ly6G was administered via i.p. injection with 200 µg every other day. Rat IgG1 (BioXCell; clone

Bellomo G, *et al. Gut* 2022; 71:2284–2299. doi: 10.1136/gutjnl-2021-325272

HRPN) was used as isotype control in anti-CSF1 study and Rat IgG2 (BioXCell; clone 2A3) was used as isotype control in the neutrophil depletion studies and in the anti-CSF1R study, respectively. In one survival study tumour bearing untreated and gemcitabine-treated mice were randomized to receive warfarin dissolved at a concentration of 0.5 mg/L in drinking water, from day 7 onwards. The water bottles containing warfarin were replenished every other day.

Liver experimental micro-metastases were induced by implanting 5 x 10^5 $KPC^{luc/zsGreen}$ FC1199 in 25 µL of PBS into the spleen of immunocompetent isogenic C57BL/6 mice using a Hamilton 29 G syringe. For selected experiments, macrophages, neutrophils or CD8+ T cells were depleted using colony-stimulating factor 1 (CSF-1) –neutralizing antibody (BioXCell, clone 5A1), Ly6G-neutralizing antibody (BioXCell, clone 1A8) or CD8-neutralizing antibody (clone 2.43) respectively; in one experiment anti-Ly6G and anti-CD8 were used in combination. Anti-CSF1 was administered via i.p. injection with an initial dose of 400 µg and subsequent doses 6 days a part containing 200 µg. Anti-Ly6G was administered via i.p. injection with 200 µg every other day. Anti-CD8 was administered via i.p injection with 200 µg every other day. Rat IgG1 (BioXCell; clone HRPN) was used as isotype control in the anti-CSF1 macrophage depletion studies, Rat IgG2 (BioXCell; clone 2A3) was used as isotype control in anti-Ly6G neutrophil depletion studies and Rat IgG2b (BioXclone LTF-2) in the anti-CD8T cell depletion studies, respectively. Tumour bearing mice were treated with gemcitabine-HCl (100 mg/kg) 3 days post cancer cell implantation via i.p. injection. In one experiment, mice were treated with FOLFIRINOX (Oxaliplatin 2.5 mg/kg, Fluorauracil 25 mg/kg, Irinotecan 25 mg/kg, Folinic acid 50mg/ml) via i.v. or gemcitabine-Nab Paclitaxel (gemcitabine 50 mg/kg via i.p., Abraxane 50 mg/kg via i.v), 5 days post cancer cell implantation. Oxaliplatin, Fluorauracil, Irinotecan were kindly provided by the Liverpool Clatterbridge Cancer Centre. In one experiment gemcitabine-treated mice were dosed with SB225002 (iCXCR2, CXCR2 inhibitor, Sigma, SML0716) starting from day7 via i.p. at a dose of 10 mg/kg daily. In one experiment, untreated and gemcitabine-treated mice were randomized to receive warfarin dissolved at a concentration of 0.5 mg/L in drinking water, from day 7 onwards. The water bottles containing warfarin were replenished every other day. In multiple experiments, untreated and gemcitabine-treated mice and neutrophils-depleted gemcitabine- treated mice were treated with Bemcentinib (R248) (MCE, HY-15150), starting from day 7 post cancer cell implantation until the endpoint. Mice were administered twice per day with 7 µg of R248, dissolved in 5% DMSO 95% corn oil, via oral gavage.

Bellomo G, *et al. Gut* 2022; 71:2284–2299. doi: 10.1136/gutjnl-2021-325272

Syngeneic orthotopic pancreatic cancer model

Primary tumour formation was induced by implanting 2 × 10^5 KPC$^{luc/zsGreen}$ FC1245 in 30 µL of Matrigel (VWR, 734-0269) into the pancreas of immune-competent syngeneic C57Bl/6 6- to 8-week-old female mice using a Hamilton 29 G syringe. Tumour bearing mice were treated with gemcitabine-HCl (100 mg/kg) 8 days post cancer cells implantation via i.p. injection. Untreated and gemcitabine-treated mice were randomized to receive Warfarin dissolved at a concentration of 0.5 mg/L in drinking water from day 12 until endpoint. The water bottles containing warfarin were replenished every other day.

Autochthonous model of pancreatic cancer KPC

KPC mice on a mixed background were bred in-house at the CRUK Beatson Institute and maintained in conventional caging with environmental enrichment, access to standard chow and water *ad libitum*. All animal experiments were performed under a UK Home Office licence and approved by the University of Glasgow Animal Welfare and Ethical Review Board. Genotyping was performed by Transnetyx (Cordoba, TN, USA). KPC mice of both sexes were treated with gemcitabine-HCl in saline (100 mg/kg). Mice were monitored 3 times weekly and palpable tumour bearing mice were recruited onto study. Vehicle and gemcitabine were administered by intraperitoneal injection every 3 days. Mice were checked 3 times per week and culled and tissues harvested at humane endpoint.

Metastatic tumour burden quantification

Liver metastasis and spleen tumour burden was quantified, *in vivo* and *ex vivo*, by assessing bioluminescence signal (BLI; IVIS, Perkin Elmer) generated by KPC$^{luc/zsGreen}$ cells. For *in vivo* detection, the BLI signal was measured post i.p. injection of Beetle luciferin (Promega; 3 mg/mouse) and quantified as total flux (photons/sec). Metastases-bearing mice were subject to *in vivo* BLI prior treatment initiation to quantify the change in tumour burden. For *ex vivo* investigation, livers and spleens were surgically removed, covered with luciferin and bioluminescence signal measured. Total flux were quantified using Living Image software v4.5 (Perkin Elmer). In some experiments, mice were euthanized at indicated time points and metastatic tumour burden was assessed by quantifying the size of metastatic lesions in haematoxylin and eosin-stained paraffin-embedded liver sections. Zeiss Axio Observer Light Apotome.2 Microscope and Zeiss ZEN imaging software were used.

Bellomo G, *et al. Gut* 2022; 71:2284–2299. doi: 10.1136/gutjnl-2021-325272

Human studies

Human studies using blood and liver tissue samples were approved by the National Research Ethics (NRES) Service Committee North West – Greater Manchester REC15/NW/0477.

Human blood samples were obtained from healthy donors or from advanced PDAC patients with liver metastasis (all pathologically confirmed) undergoing their first cycle of gemcitabine treatment (one treatment/week during 3 weeks (week 0, 1, 2), followed by two weeks rest (week 4). Blood samples were collected at the following two time points: prior to treatment (baseline, week 0) and week 4. Blood was collected in EDTA coated tubes and immediately processed for neutrophils isolation. Haematology blood results were provided by the Liverpool Clatterbridge Cancer Centre for the following three time points: baseline, week 1 and week4.

Liver biopsies were collected from treatment-naïve advanced PDAC patients with liver metastasis (all pathologically confirmed). Liver biopsies from patients undergoing surgical resection of colorectal liver metastases (treatment naïve and following neoadjuvant chemotherapy with oxaliplatin, FOLFOX, and/or capecitabine) were analysed following appropriate ethical approval (REC 15/NW/0477). All individuals provided informed consent for blood or tissue donations on approved institutional protocols.

In vitro chemotherapeutic treatment

KPCs and Panc1 cells were seeded in petri dishes (d = 10 cm). At 70-80% confluence, murine and human cells were washed in PBS and treated with 100 nM or 200 nM gemcitabine-HCl, respectively, in DMEM + 2% FBS, for 24 hours.

CellTiter-Glo® luminescent cell viability assay

KPC cells were seeded as previously described and treated with 10, 20, 50, 100 and 200 nM of gemcitabine-HCl for 24 or 48 hours. Cell viability was assessed by CellTiter-Glo® assay according to manufactures (Promega, G9241).

Preparation of tumour conditioned media

Bellomo G, *et al. Gut* 2022; 71:2284–2299. doi: 10.1136/gutjnl-2021-325272

For generating tumour conditioned media (TCM) from gemcitabine-treated cells (TCM-gem), cells were cultured to reach 70-80% confluency. Murine and human cell lines were washed 3 times with PBS before addition of 100 nM and 200 nM gemcitabine, respectively in DMEM containing 2% FBS for 24 hours. After gemcitabine treatment, cells were washed 3 times with PBS and incubated with serum free media for 24 hours. The supernatant was harvested and filtered to remove debris and dead cells using a 0.22 µm syringe filter (Fisher Scientific). The control TCM (TCM-Ctr) was generated by seeding cancer cells and allowing them to grow to 70-80% confluency. KPC FC1199 and Panc1 were incubated with 2 % FBS DMEM for 24 hours; cells were washed and cultured with serum free DMEM for 24 hours. The supernatant was harvested and filtered to remove debris and dead cells using a 0.22 µm syringe filter (Fisher Scientific).

Nanostring technology

Pieces of <3 mm^3 of metastatic liver were submerged in 10 volumes of RNAlater (Qiagen). Extraction of total RNA was performed using the RNeasy Mini kit (Qiagen), following the manufacturer's instructions. The RNA concentrations of samples were measured using a NanoDrop 2000 Spectralphotometer (Thermo Fisher); all RNA samples used in this study exhibited optical density (OD) 260/280 ratios between 1.9 and 2.1 and OD 260/230 ratios above 1.8. A total of 770 immune-related mouse genes were analysed using the nCounter Mouse PanCancer Immune Profiling Panel (NanoString). Data analyses were performed using the manufacturer specific software nSolver™ version 4.0 with the nCounter Advanced Analysis plugin version 2.0. To analyse the activity of regulated pathways, we used pathway scoring with default settings as provided by the software. Pathway scores calculated by the software plugin are based on a sample's gene expression profile using the first principal component analysis of a gene set data, with a positive score corresponding to increased expression of more than half its genes.

RNA hybridization

RNA *in situ* hybridization (RNAScope, ACD Bio-Techne) was performed on formalin-fixed paraffin-embedded sections using the RNAScope 2.5 HD Detection Kit RED according to manufacturer's instructions. The probe used was directed against mouse Gas6 (cat no 473831) or human Gas6 (cat no 427811). The images, taken using Zeiss Axio Observer Light Apotome.2 Microscope, were analysed trough the software QuPath for automated analysis by assigning a staining score for Gas6 of *low*, *medium* or *high* to each cell

Bellomo G, *et al. Gut* 2022; 71:2284–2299. doi: 10.1136/gutjnl-2021-325272

according to the staining intensity (intensity threshold parameters: *low* ≤ 0.27, *medium* = 0.57-0.77, *high* ≥ 0.78).

Flow cytometry and cell sorting

Single-cell suspensions from murine metastatic liver tumours were prepared by first selecting macroscopically tumour-rich areas in the liver followed by mechanical and enzymatic disruption in Hanks Balanced Salt Solution (HBSS) with 1 mg/mL Collagenase P (Roche) as previously described [2, 4]. In some occasions, tumour free lungs were resected and processed using the same methods. Briefly, cell suspensions were centrifuged for 5 minutes at 1,200 rpm, resuspended in HBSS, and filtered through a 500 µm polypropylene mesh (Spectrum Laboratories). Cell suspensions were resuspended in 1mL 0.05% trypsin and incubated at 37 °C, for 5 minutes. Cells were filtered through a 70 mm cell strainer (Miltenyi) and resuspended in 0.5% BSA/PBS. Peripheral blood was obtained by performing tail nick and collected in EDTA coated tubes (Sarstedt). Red blood cells were lysed using 1x Red blood cell lysis buffer (Biolegend). Cells were resuspend in 2% BSA/PBS, blocked for 10 minutes on ice with Fc block (BD Pharmingen; clone 2.4 G2) and stained with Sytox-blue viability marker (Life Technologies) and conjugated antibodies against CD45 (clone 30F-11), F4/80 (clone BM8), CD206 (clone C068C2), MHCII (clone M5/114.15.2), CD86 (clone GL-1), CD3ε (clone 145-2C11), CD8α (clone 53-6.7), CD4 (clone RM4-5), NK-1.1 (clone PK136), CD69 (clone H1.2F3), CD11b (clone M1/70), Ly6C (clone HK 1.4), Ly6G (clone 1A8), CXCR2 (clone SA044G4), CD103 (clone 2E7), CD11c (clone 418) and FoxP3 (clone Mf-14) (all purchased from Biolegend). Intracellular staining of FoxP3 staining was performed after surface staining; cells were fixed and permeabilised using FOXP3 Fix/Perm Buffer Set (Biolegend) according to manufacture instructions. Human liver biopsies were collected in cold PBS tubes and processed as previously described for obtaining single cell suspensions. Cells were resuspended in 2% BSA/PBS, blocked using human TrueStain FcX (Biolegend) and then stained with Sytox-blue viability marker (Life Technologies) and conjugated antibodies against CD45 (clone HI30), CD11b (clone M1/70), CD14 (clone HCD14), CD15 (clone HI98), CD16 (clone 3G8) epCAM (clone 9C4), HLA-DR (clone L243) and PDGFR-beta (clone 18A2).

Flow cytometry was performed on a FACS Canto II (BD Biosciences), and fluorescence-activated cell sorting (FACS) was carried out using FACS Aria IIIu (BD Biosciences). Cells were sorted directly in RLT buffer + β-mercaptoethanol according to the manufacturer's instruction for RNA isolation (Qiagen), or in MACS buffer or DMEM supplemented with 20% FBS for *in vitro* assays.

Bellomo G, *et al. Gut* 2022; 71:2284–2299. doi: 10.1136/gutjnl-2021-325272

Analysis and quantification of immune cells in liver metastasis by mass cytometry

Single-cell suspensions from murine metastatic liver tumours were prepared as described above. Suspensions were further enriched for immune cells by density gradient centrifugation using Histopaque-1083 (Sigma Aldrich) at 400x g for 30 minutes at room temperature without brakes. The cloudy band/interface containing the cells plus the bottom layer was transferred into new tube and gently washed with PBS. After one wash with PBS, the cell suspension was washed in double-deionised water (ddH2O; ≥ 18Ω)/Maxpar cell staining buffer (1:2 dilution). The pellet was resuspended in 1 ml of Maxpar cell staining buffer and cells were stained with Cell-ID 195-Cisplatin (Fluidigm) viability marker in Maxpar PBS (Fluidigm) for 5 min. Cells were washed with Maxpar cell staining buffer, blocked with Fc Block (BD Pharmingen, Clone 2.4G2) on ice for 10 min and metal-conjugated antibody cocktail added and incubated for 40 min at 4°C. The antibody cocktail contained ^{141}Pr-Ly6G (clone 1A8), ^{142}Nd-CD11c (clone N418), ^{143}ND-CD69 (clone H1.2F3),^{146}Nd-F4/80 (clone BM8), ^{147}Sm-CD45 (clone 30-F11), ^{148}Nd-CD11b (clone M1/70), ^{149}Sm-CD19 (clone 6D5), ^{152}Sm-CD3ε (clone 145-2C11), ^{162}Dy-Ly6C (clone HK1.4), ^{168}Er-CD8α (clone 53-6.7), ^{169}Tm-TCRb (clone H57-597), ^{170}Er-NK1.1 (clone PK136), ^{172}Yb-CD86 (clone GL1), ^{174}Yb-MHC II (clone M5/114.15.2) and ^{176}Yb-B220 (clone RA3-6B2). Antibodies were used at the concentrations recommended by the manufacturer. Cells were then washed twice in cell staining buffer and stained with 125 μM Intercalator-^{191}Ir (Fluidigm) diluted 1:2,000 in Maxpar fix and perm buffer (Fluidigm) overnight at 4°C. Cells were then washed twice in Maxpar cell staining buffer followed by two washes in 18Ω distilled water (Fluidigm) and resuspended in 0.1X EQ^{TM} Four Element Calibration Beads (Fluidigm) prior acquisition on the Helios CyTOF system (Fluidigm). Samples were acquired at a rate of <500 events/sec. All generated FCS files were normalized and EQ beads standard [5]. Data analysis was performed using Cytobank software (mrc.cytobank.org, v6.3 and v7.0, Beckman Coulter); manual gating was used to remove debris, identify single cells ($^{191}Ir^+$) and to distinguish between dead cells ($^{195}Pt^+$). viSNE analysis was performed on the data utilizing t-stochastic neighbour embedding (t-SNE) mapping based on high dimensional relationships. Viable CD45+ singlets selected by manual gating were used for viSNE unsupervised clustering using equal sampling. Manual gating was then performed on the viSNE map created to determine cell population percentages.

RNA Sequencing

Bellomo G, *et al. Gut* 2022; 71:2284–2299. doi: 10.1136/gutjnl-2021-325272

Murine neutrophils were isolated from metastasis bearing livers by FACS as described in the previous section. Cells were lysed in RNAlater buffer (Qiagen) and total RNA purification was performed with the RNeasy Kit (Qiagen). Total RNA integrity was assessed using Qubit and Bioanalyzer from Agilent. RNA-Seq libraries were prepared using the NEBNExt polyA selection and Ultra Directional RNA library preparation kit following manufactures. Libraries were PCR amplified and sequenced on an Illumina HiSeq 400 System, generating 150 bp reads. Reads were aligned and gene level counts generated using STAR 2.7.2b using mouse genome 'GRCm38' downloaded from Illumina igenomes [6]. Quality control metrics were checked using FastQC [7]. Genes with counts < 10 were removed and the data was normalised for differences in sequencing depth using the robust fragments per million (FPM) method available in the R package DESeq2[8]. Genes were annotated using KEGG pathways or Gene Ontology terms using the relevant annotation packages available within BioConductor. Normalised fragments per million mapped fragments (FPM) count data were obtained for a total of 12,651 genes. Those with a mean FPM of <1 were filtered out, as were genes with counts for only one condition (control [CTR] or Gem-treated [CTX]), creating a dataset with 9,616 differentially-expressed genes. Genes annotated with the Gene Ontology (GO) Cellular Component terms "cell surface" (GO:0009986) and/or "extracellular region" (GO:0005576) and GO Molecular Function (GOMF) term "receptor ligand activity" (GO:0048018) were extracted for further analysis, to generate supplementary tables 1 and 2.

Magnetic bead isolation of human and murine neutrophil and murine macrophages

Murine neutrophils were isolated from liver and spleen, macrophages were isolated from liver. Liver samples were prepared as described above for preparation of flow cytometry samples and Ly6G$^+$ neutrophils and F4/80$^+$ macrophages were isolated by positive selection, according to the manufacturer's instructions (Miltenyi). Dissected spleens were dissociated in MAC buffer and passed through a 70 µm cell strainer to obtain a single-cell suspension. Cells were centrifugated (400 x *g*), and red blood cells were lysed using 1X Red blood cell Lysis buffer (Biolegend). Single cell suspensions were stained, and Ly6G$^+$ cells were isolated by positive selection, according to the manufacturer's instructions (Miltenyi). Where human neutrophils were isolated from whole blood, blood was collected in EDTA coated tubes and immediately processed for neutrophil isolation using MACSxpress® Whole Blood Neutrophil Isolation Kit (Miltenyi) according to manufacturer's protocol.

Bellomo G, *et al. Gut* 2022; 71:2284–2299. doi: 10.1136/gutjnl-2021-325272

In vitro **colony formation assay**

$KPC^{luc/zsGreen}$ and parental KPC FC1199 and Panc1 cells were seeded and treated with gemcitabine as described in the previous paragraph. Tumour cells were then washed in PBS and serum starved in DMEM, for 24 hours, except for $KPC^{luc/zsGreen}$ which were incubated in 2%FBS/DMEM and 100nM gemcitabine for 24 hours. A 24-well or 48-well plate was coated with a layer of 0.5% agar solution made of phenol-free DMEM without FBS. Tumour cells were strained through a 70µm cell filter to ensure a single cell suspension. The cells were then embedded at a concentration of 10000 or 40000 cells/well in a 0.3% agar mix consisting of phenol-free DMEM supplemented with 2% FBS. A layer of 2% FBS phenol-free DMEM was added on top of the agar matrix. In one experiment, tumour cells were incubated with neutrophils ($Ly6G^+$ cells) or macrophages ($F4/80^+$ cells), at a ratio 1:1. Liver metastasis derived neutrophils or macrophages were MACS-sorted (as described in previous section from untreated ($Ly6G_{CTR}$ or $F4/80_{CTR}$) or gemcitabine treated ($Ly6G_{CTX}$ or $F4/80_{CTX}$) tumour bearing mice and co-cultured with cancer cells for 6 days. In another experiment, the system was supplemented with neutralising anti-gas6 antibody (R&D Systems, AB885). Colony quantification was performed after 5 days, by measuring bioluminescence signal (IVIS, Perkin Elmer) following the addition of 10 µL of Beetle luciferin stock (1:100, final concentration 150µg/mL).

In some experiments, KPC parental FC1199 and Panc1cells were embedded with the addition of 400 ng/ml rGas6 (R&D Systems, 885-GSB-050); rGas6 was added to DMEM top layer and replaced every other day. The colonies were fixed and stained with 0.05% crystal violet in methanol/PBS and the number of colonies per well was counted at day 14 after cell seeding by bright-field microscopy.

Transwell migration assay

1 x 10^6 human or murine neutrophils were added in serum free DMEM in Transwell inserts with 5 µm pore size (Corning) and allowed to migrate to the bottom chamber of the 24-well plate for 15 hours. In one experiment, the bottom chamber was loaded with serum free DMEM +/- rCXCL2 (Peprotech, 250-20A) at a concentration of 50 ng/ml. In another experiment, the bottom chamber was loaded with tumour conditioned media generated from untreated (TCM-ctr) or gemcitabine-treated (TCM-gem) murine or human cancer cell lines (KPC or Panc1) in the presence or absence of SB225002 (iCXCR2, CXCR2 inhibitor, Sigma, SML0716) at a concentration of 25 µg/ml. Migrated neutrophils were recovered from the lower chamber and counted by using a haemocytometer.

Precision cut liver slicing and *ex vivo* culture

Precision cut liver slicing was performed as previously reported [9, 10]. Briefly, PDAC patient liver biopsies were embedded in 3% UltraPureLMP Agarose (Invitrogen, 16520050) dissolved in PBS, onto specimen small dishes; sectioning was performed using Leica vibrating blade microtome VT1200 S (Leica, Wetzlar, Germany), using stainless steel razor blades (Personal Medical, Staunton, VA, USA) under buffered conditions with ice-cold HBSS containing 25 mM glucose (Merck, Darmstadt, Germany), at the following adjustable settings: knife angle: 15°; sectioning speed: 0.4–1 mm/s; oscillation amplitude: 3 mm; step size: 200 lm; retract: 10 lm; continuous stroke.

After sectioning, liver slices (thickness: 200-300 nm) were cultured on top of Nucleopore Track-Etch Membrane (GE Healthcare Life Science, 10417301) to facilitate oxygenation, in 24-well plate in William's E medium (Sigma) supplemented with 25 mM glucose, 20 mM L-Glutamine (Sigma), 1% Pen Strep (Gibco) and 100 mM gemcitabine, at 37°C for 24 hours. Liver slices were washed in PBS and the media was replaced with William's E medium, supplemented with glucose, Pen Strep and with or without rGas6 at a concentration of 400 ng/ml, incubated at 37°C for 24 hours. During the two-day culture the media was replaced every 24 hours.

RT-qPCR

Total RNA purification was performed with the RNeasy Kit (Qiagen), and cDNA was generated using the QuantiTect Reverse Transcription Kit (Qiagen) according to manufacturer's instructions. 500 ng of total RNA was used to generate cDNA. qPCR was performed using 5 x HOT FIREPol EvaGreen qPCR Mix Plus (ROX; Solis Biodyne) on an MX3005P instrument (Stratagene). Three-step amplification was performed (95°C for 15 seconds, 60°C for 20 seconds, and 72°C for 30 seconds) for 45 cycles. Relative expression levels were normalized to Gapdh expression according to the formula: 2^-(Ct gene of interest – Ct Gapdh). Fold increase in expression levels was calculated by the comparative Ct method: 2^ (-ddCt). The following QuantiTect Primers Assays were used to assess mRNA levels: Mm-Gapdh (Mm_Gapdh_3_SG; QT01658692), Mm-Cxcl-1 (Mm_Cxcl1_1; QT00115647), Mm-Cxcl2 (Mm_Cxcl2_1; QT00113253), Mm-Cxcl-5 (Mm_Cxcl5_2; QT01658146), Mm-Gas6 (Mm_Gas6_1_SG; QT00101332), Hs-Gapdh (Hs_Gapdh_1_SG; QT00079247), Hs-Gas6 (Hs_Gas6_1_SG; QT00049126) were purchased from Qiagen. Hs-Cxcl1 (Hs_Cxcl1_Fw 5'-AGCTCTTCCGCTCCTCTCA), Hs-Cxcl2 (Hs_Cxcl2_Fw 5'-GAAAGCTTGTCTCAACCCG), Hs-Cxcl5 (Hs_Cxcl5_Fw 5'-CCAGTAGTTAGCTTTCTTCCTGGAT), Hs-Cxcl8 (Hs_Cxcl8_Fw 5' CCTTCCTGATTTCTGCAGCTC) were purchased from Sigma-Aldrich.

Immunohistochemistry analysis

Deparaffinization and antigen retrieval were performed using an automated DAKO PT-link. Paraffin-embedded human and mouse liver metastatic sections were immunostained using the DAKO envision system-HRP. Tissue sections were incubated overnight at 4°C with primary antibodies: Cytokeratin 19 (Abcam, ab53119, 1:100); MUC-1 (Abcam, ab89492, 1:100); Ki67 (Abcam, ab15580, 1:1000); CC3 (Cell Signalling, #9661, 1:300); P-AXL (R&D System, AF2228); MPO (Abcam, ab9535, 1:100); YM-1 (StemCell Technology, 60130, 1:200); NKp46 (R&D System, AF2225). Secondary HRP-conjugated antibodies were incubated for 30 minutes at room temperature. Staining was developed using diaminobenzidine and counterstained with haematoxylin. Tumour cells in the liver were identified by their characteristic formation of ductal structures during metastatic growth.

Immunofluorescence analysis

Murine liver tissues were fixed using a sucrose gradient method to preserve the zsGreen fluorescence. Briefly, livers were fixed in 4% Formaldehyde + 10% sucrose in PBS for 4 hours and then transferred to 20% sucrose in PBS for 8-10 hours. Tissues were transferred into 30% sucrose for an additional 8-10 hours, embedded in OCT medium and stored at -80°C.

For immunofluorescence staining, 5 mm liver sections were permeabilized by 0.1% TritonX-100 (Sigma Aldrich) for 10 minutes. Unspecific bindings were prevented by using PBS + 8% normal goat serum for 1 hour at room temperature. Tissue sections were incubated overnight at 4°C with the following antibodies: CD8 (BioLegend, 100701, 1:400); CC3 (Cell Signalling Technologies, 9661); F4/80 (BioLegend, 123102, 1:100); iNOS (Abcam, ab15323, 1:50); MUC-1 (Abcam, ab89492, 1:100); Ki67 (Abcam, ab15580, 1:1000); MPO (Abcam, ab9535, 1:50). The next day, tissue sections were washed in PBS and stained with the secondary antibody goat anti-rabbit conjugated to anti-mouse IgG Alexa Fluor(AF)-488 (Abcam, ab150105, 1:200); anti-rabbit IgGAF-488 (Abcam, ab150077, 1:300); anti-rat IgG AF-594 (Abcam, ab150160, 1/300); anti-rabbit IgG AF-594 (Abcam, ab150080, 1/200) and DAPI (Life Technologies, 1:500) for 1 hour at room temperature. Luc/zsGreen-transfected cells were detected by their intrinsic signal. For CD8 staining VECTASTAIN® Elite® ABC-HRP Kit (Peroxidase, Rat IgG) (Vector laboratories) was used. TUNEL staining was performed according to manufacturer's instruction (Thermofisher, 15313038). Sections were finally mounted using Dako Fluorescent Mounting Medium. All tissue sections were imaged using an Axio Observer Light Microscope with the Apotome.2 (Zeiss) and quantified using the Zen Software (Zeiss).

Bellomo G, *et al. Gut* 2022; 71:2284–2299. doi: 10.1136/gutjnl-2021-325272

Statistical analysis

Statistical significance (analysed with GraphPad Prism v5 or v8 software) was determined using two-tailed unpaired Student's t tests when comparing differences between two experimental groups. Unless otherwise stated, for multiple comparisons, one-way ANOVA with Bonferroni was used for all experiments with more than two groups. For Figure 2D, E one-way ANOVA with Dunnett's *post hoc* testing was performed. For Figures 4G, 4J, 8D and Supplementary Figure S5F, N two-way ANOVA with Bonferroni was used. The Log-rank (Mantel-Cox) test was used to assess differences in survival for the metastatic survival study under cytotoxic treatment. A *P*-value < 0.05 was considered statistically significant and *P*-values are indicated in the figures using asterisks: * $P<0.05$; ** $P<0.01$; *** $P<0.001$; **** $P<0.0001$; ns denotes not significant.

Supplementary Table S1. Top upregulated genes GO extracellular.

Supplementary Table S2. Complete gene list GO extracellular.

Supplementary Table S3. Haematology PDAC patients.

SUPPLEMENTARY METHODS REFERENCES

1 Hingorani SR, Wang L, Multani AS, Combs C, Deramaudt TB, Hruban RH*, et al.* Trp53R172H and KrasG12D cooperate to promote chromosomal instability and widely metastatic pancreatic ductal adenocarcinoma in mice. Cancer Cell 2005;**7**:469-83.

2 Nielsen SR, Quaranta V, Linford A, Emeagi P, Rainer C, Santos A*, et al.* Macrophage-secreted granulin supports pancreatic cancer metastasis by inducing liver fibrosis. Nat Cell Biol 2016;**18**:549-60.

3 D'Costa Z, Jones K, Azad A, van Stiphout R, Lim SY, Gomes AL*, et al.* Gemcitabine-Induced TIMP1 Attenuates Therapy Response and Promotes Tumor Growth and Liver Metastasis in Pancreatic Cancer. Cancer Res 2017;**77**:5952-62.

4 Quaranta V, Rainer C, Nielsen SR, Raymant ML, Ahmed MS, Engle DD*, et al.* Macrophage-Derived Granulin Drives Resistance to Immune Checkpoint Inhibition in Metastatic Pancreatic Cancer. Cancer Res 2018;**78**:4253-69.

5 Finck R, Simonds EF, Jager A, Krishnaswamy S, Sachs K, Fantl W*, et al.* Normalization of mass cytometry data with bead standards. Cytometry A 2013;**83**:483-94.

6 Dobin A, Davis CA, Schlesinger F, Drenkow J, Zaleski C, Jha S*, et al.* STAR: ultrafast universal RNA-seq aligner. Bioinformatics 2013;**29**:15-21.

7 https://www.bioinformatics.babraham.ac.uk/projects/fastqc/.

8 Love MI, Huber W, Anders S. Moderated estimation of fold change and dispersion for RNA-seq data with DESeq2. Genome Biol 2014;**15**:550.

9 de Graaf IA, Olinga P, de Jager MH, Merema MT, de Kanter R, van de Kerkhof EG*, et al.* Preparation and incubation of precision-cut liver and intestinal slices for application in drug metabolism and toxicity studies. Nat Protoc 2010;**5**:1540-51.

10 Zimmermann M, Lampe J, Lange S, Smirnow I, Konigsrainer A, Hann-von-Weyhern C*, et al.* Improved reproducibility in preparing precision-cut liver tissue slices. Cytotechnology 2009;**61**:145-52.

SUPPLEMENTARY FIGURES

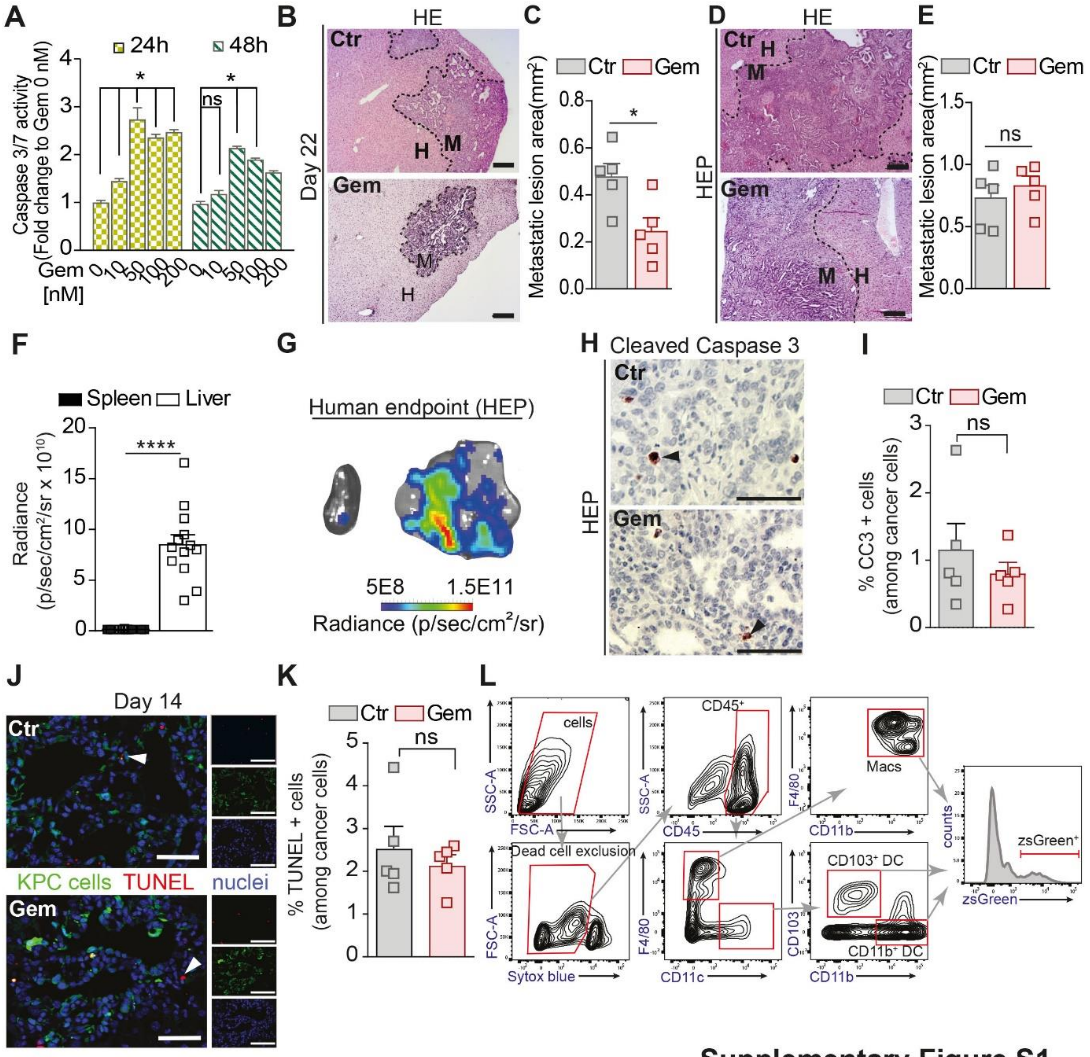

Supplementary Figure S1

Bellomo G, *et al. Gut* 2022; 71:2284–2299. doi: 10.1136/gutjnl-2021-325272

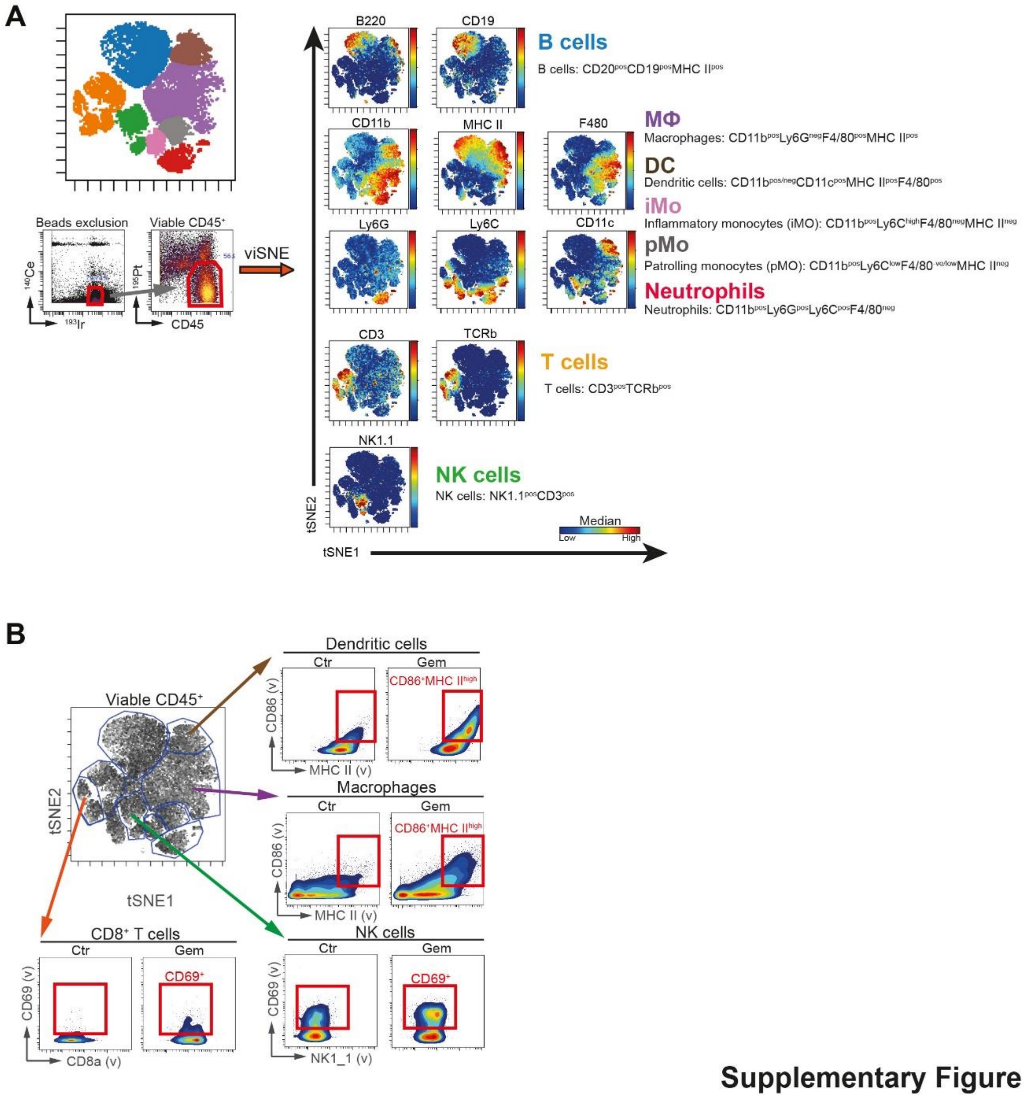

Supplementary Figure S2

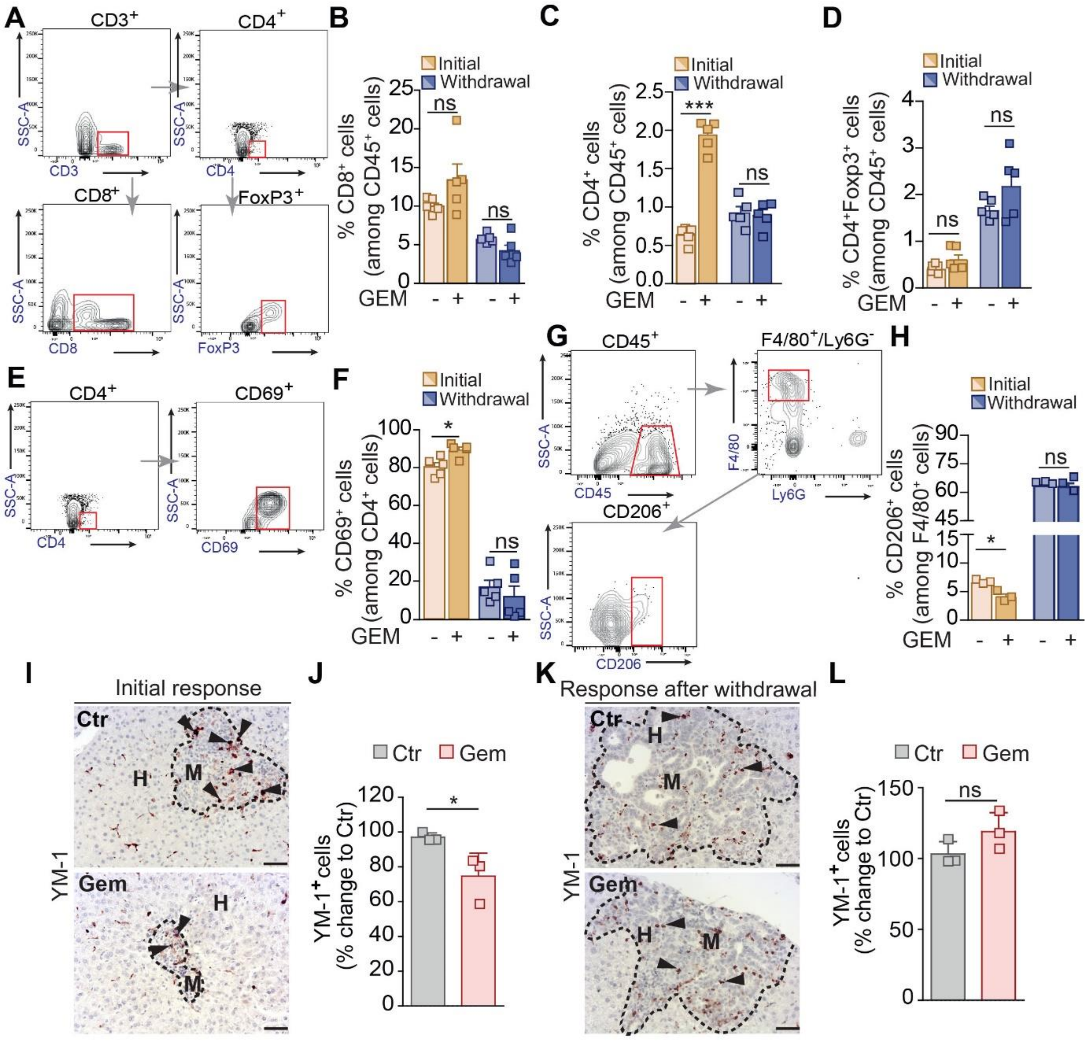

Supplementary Figure S3

Bellomo G, *et al. Gut* 2022; 71:2284–2299. doi: 10.1136/gutjnl-2021-325272

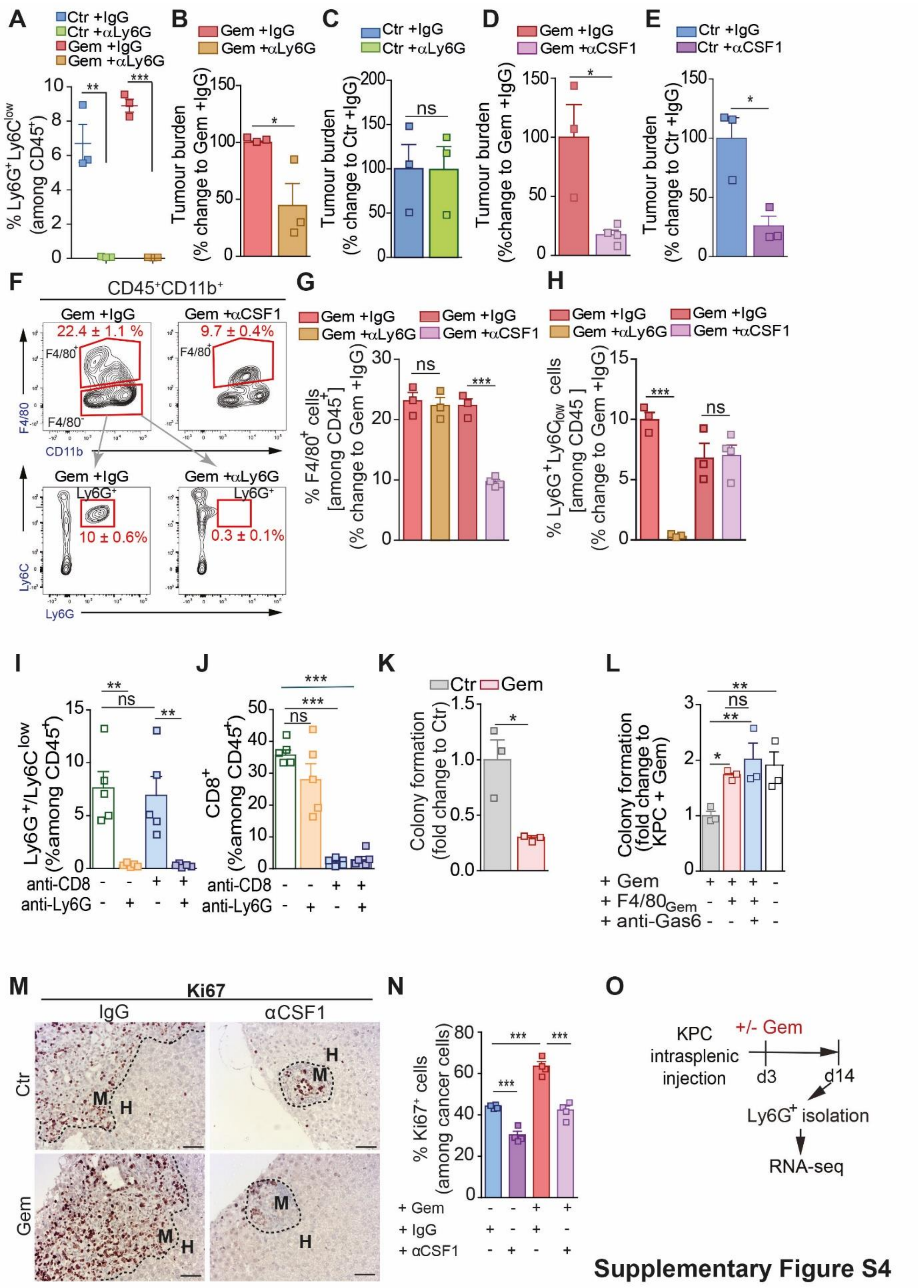

Supplementary Figure S4

Bellomo G, et al. Gut 2022; 71:2284–2299. doi: 10.1136/gutjnl-2021-325272

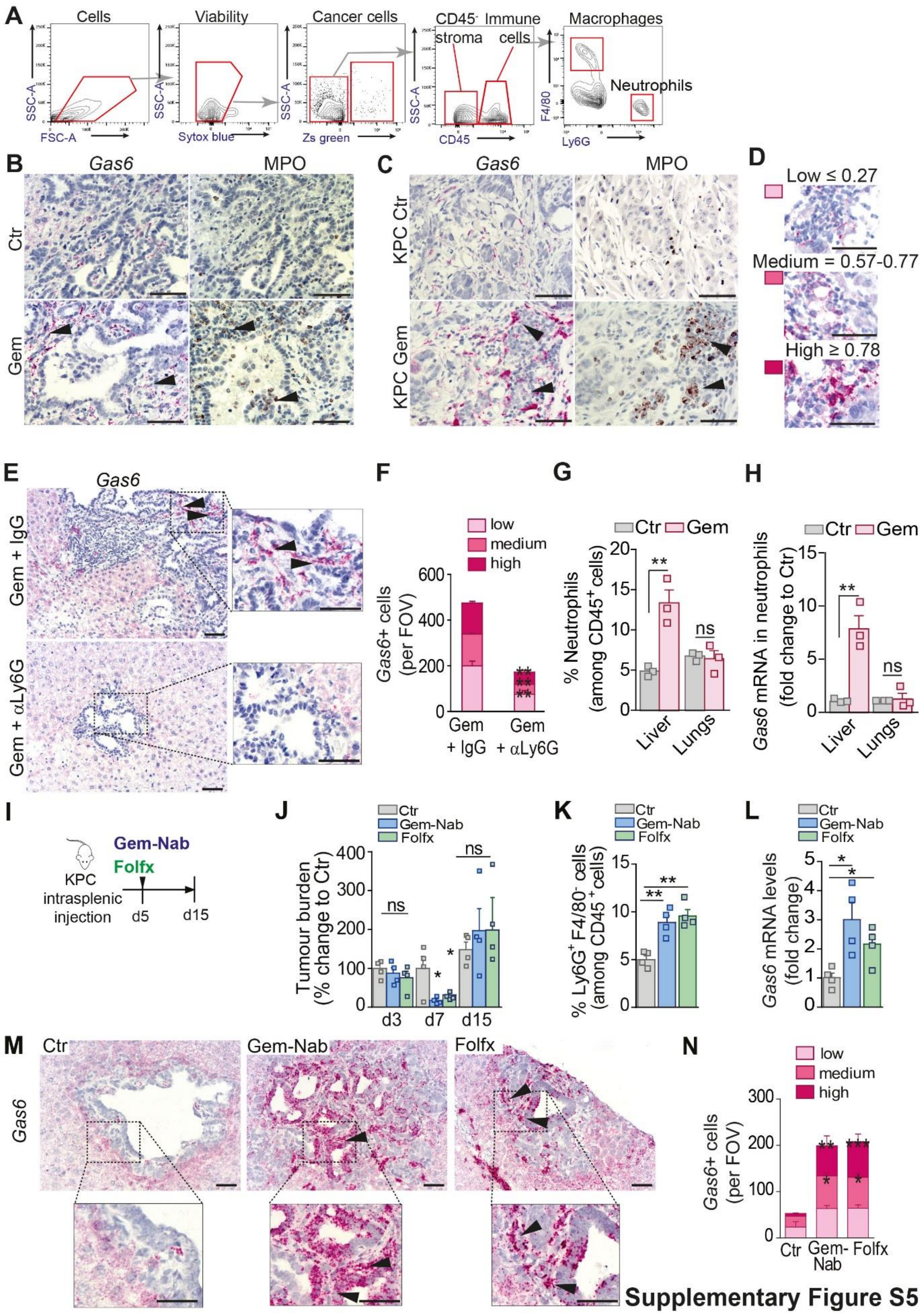

Bellomo G, et al. Gut 2022; 71:2284–2299. doi: 10.1136/gutjnl-2021-325272

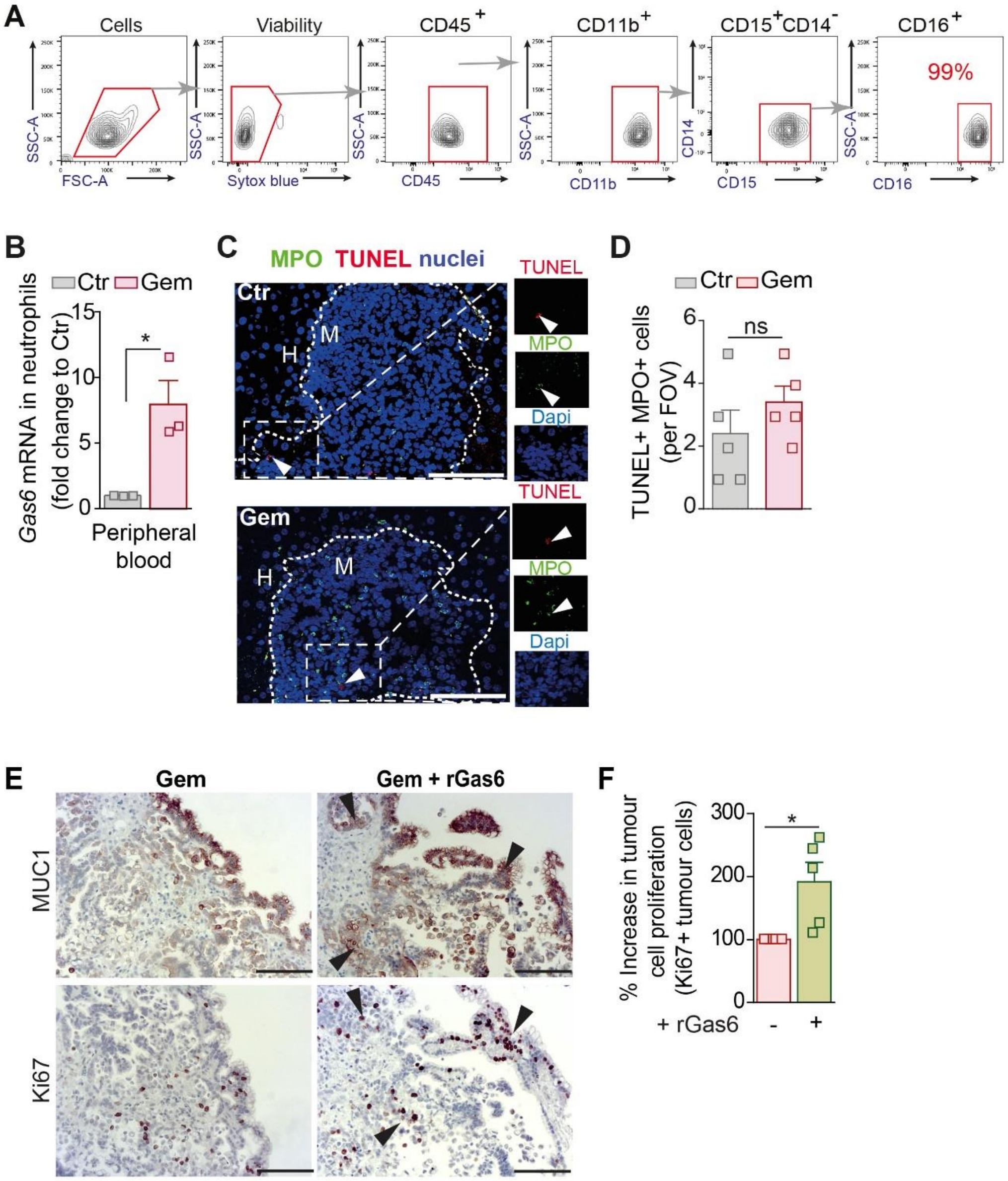

Supplementary Figure S6

Bellomo G, et al. Gut 2022; 71:2284–2299. doi: 10.1136/gutjnl-2021-325272

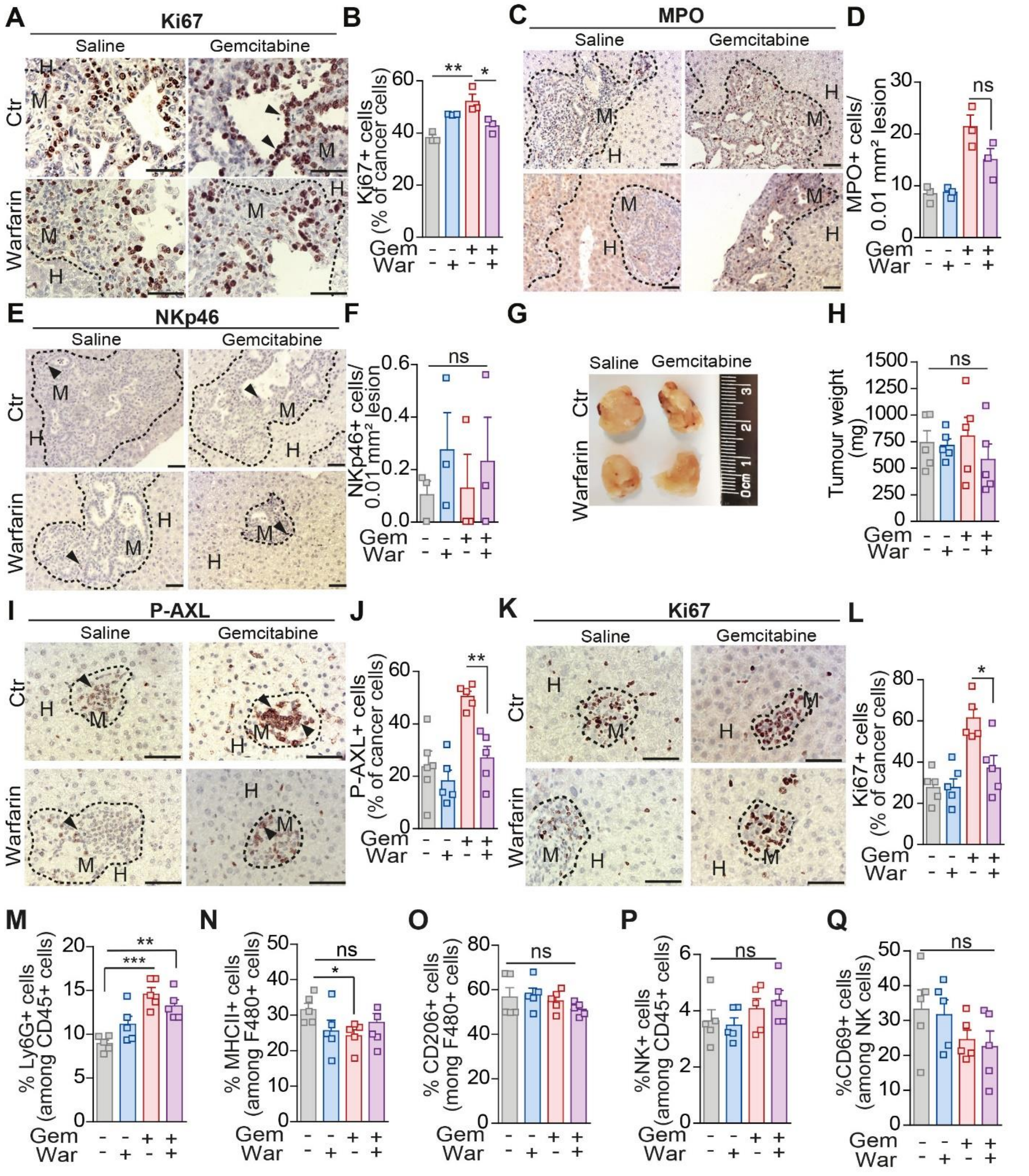

Supplementary Figure S7

Bellomo G, *et al. Gut* 2022; 71:2284–2299. doi: 10.1136/gutjnl-2021-325272

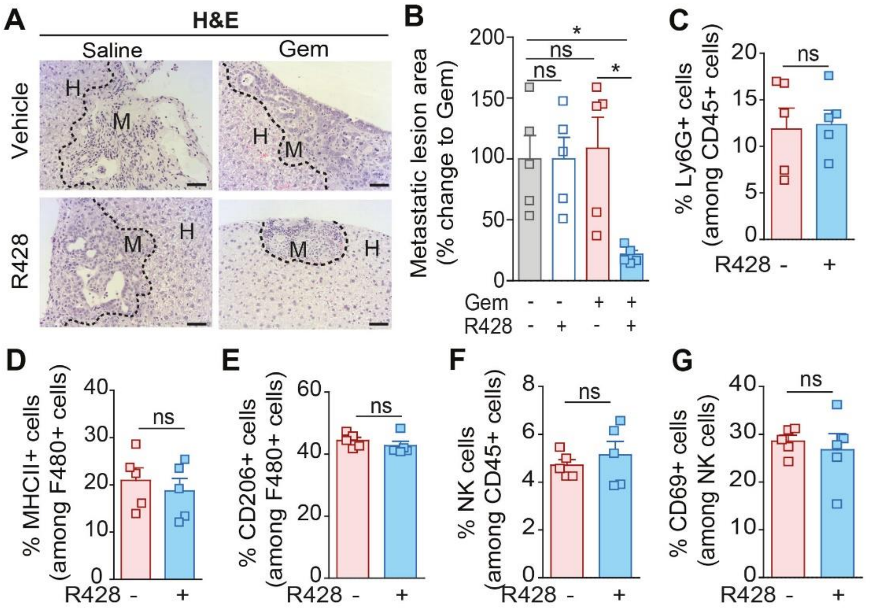

Supplementary Figure S8

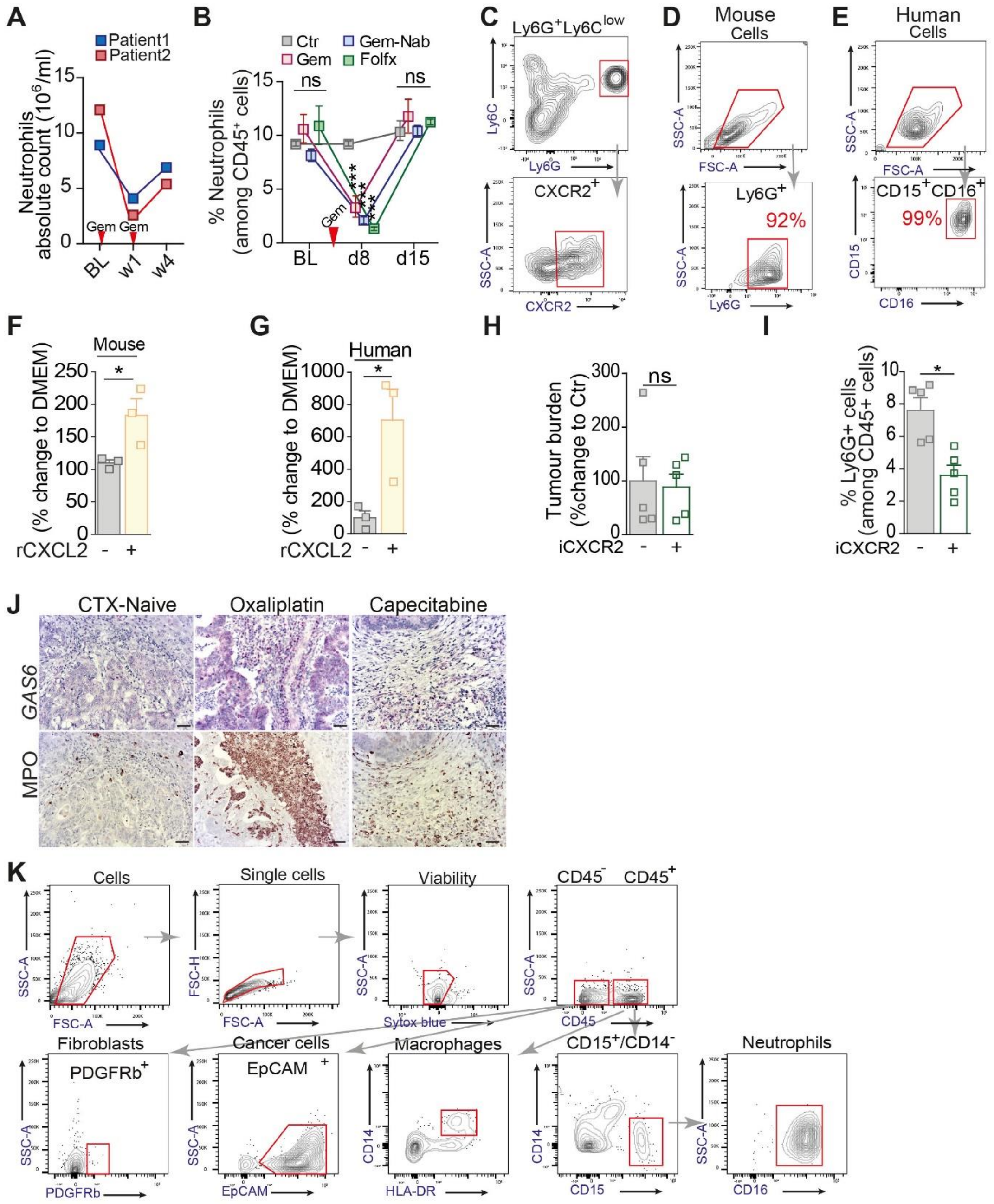

Supplementary Figure S9

Bellomo G, *et al. Gut* 2022; 71:2284–2299. doi: 10.1136/gutjnl-2021-325272
